# Supplementary material for: Secondary analysis of transcriptomes of SARS-CoV-2 infection models to characterize COVID-19
Source: Patterns (N Y). 2021 Apr 5;2(5):100247. doi: 10.1016/j.patter.2021.100247 (PMC8020120; doi:10.1016/j.patter.2021.100247)
Supplement: Document S1. Figures S1–S4 [file mmc1.pdf]

Patterns, Volume *2*

Supplemental information

Secondary analysis of transcriptomes of SARS-CoV-2 infection models to characterize COVID-19

Sudhir Ghandikota, Mihika Sharma, and Anil G. Jegga

SUPPLEMENTAL ITEMS

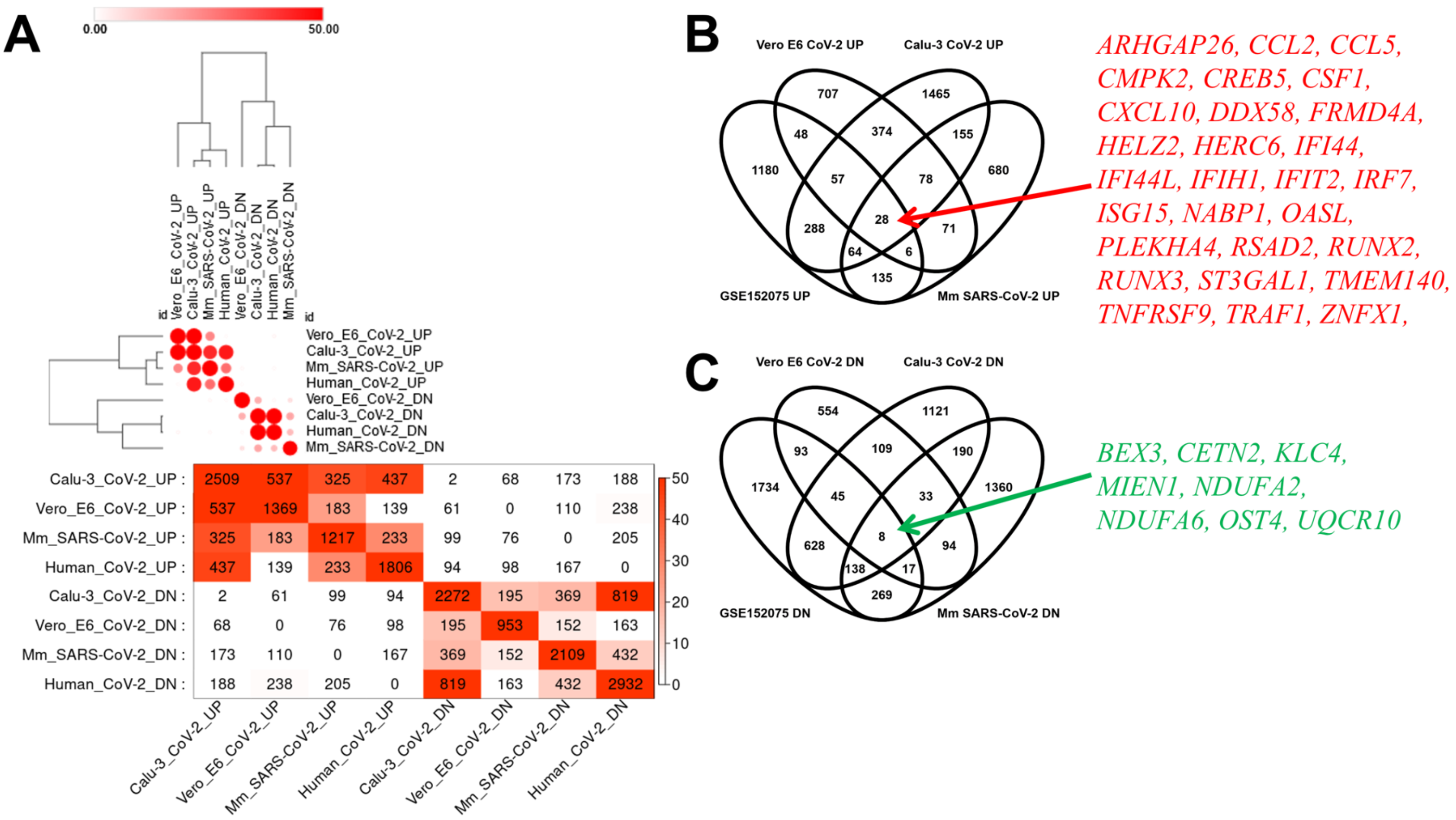

	Calu-3_CoV-2_UP	Vero_E6_CoV-2_UP	Mm_SARS-CoV-2_UP	Human_CoV-2_UP	Calu-3_CoV-2_DN	Vero_E6_CoV-2_DN	Mm_SARS-CoV-2_DN	Human_CoV-2_DN
Calu-3_CoV-2_UP :	2509	537	325	437	2	68	173	188
Vero_E6_CoV-2_UP :	537	1369	183	139	61	0	110	238
Mm_SARS-CoV-2_UP :	325	183	1217	233	99	76	0	205
Human_CoV-2_UP :	437	139	233	1806	94	98	167	0
Calu-3_CoV-2_DN :	2	61	99	94	2272	195	369	819
Vero_E6_CoV-2_DN :	68	0	76	98	195	953	152	163
Mm_SARS-CoV-2_DN :	173	110	0	167	369	152	2109	432
Human_CoV-2_DN :	188	238	205	0	819	163	432	2932

Figure S1: Transcriptomic overlap between SARS-CoV-2 infection models with that of DEGs from human nasopharyngeal swabs of COVID-19 patients. **(A)** Heatmap indicating the transcriptomic overlaps between the different SARS-CoV-2 infection models including the DEGs identified in nasopharyngeal swabs of human COVID-19 patients (GSE152075). **(B)** and **(C).** Venn diagrams showing intersections between up- or down-regulated DEGs respectively from the 3 SARS-CoV-2 models and the DEGs from nasopharyngeal swabs from human COVID-19 patients. There were 28 upregulated (Panel B) and 8 downregulated (Panel C) genes common to all.

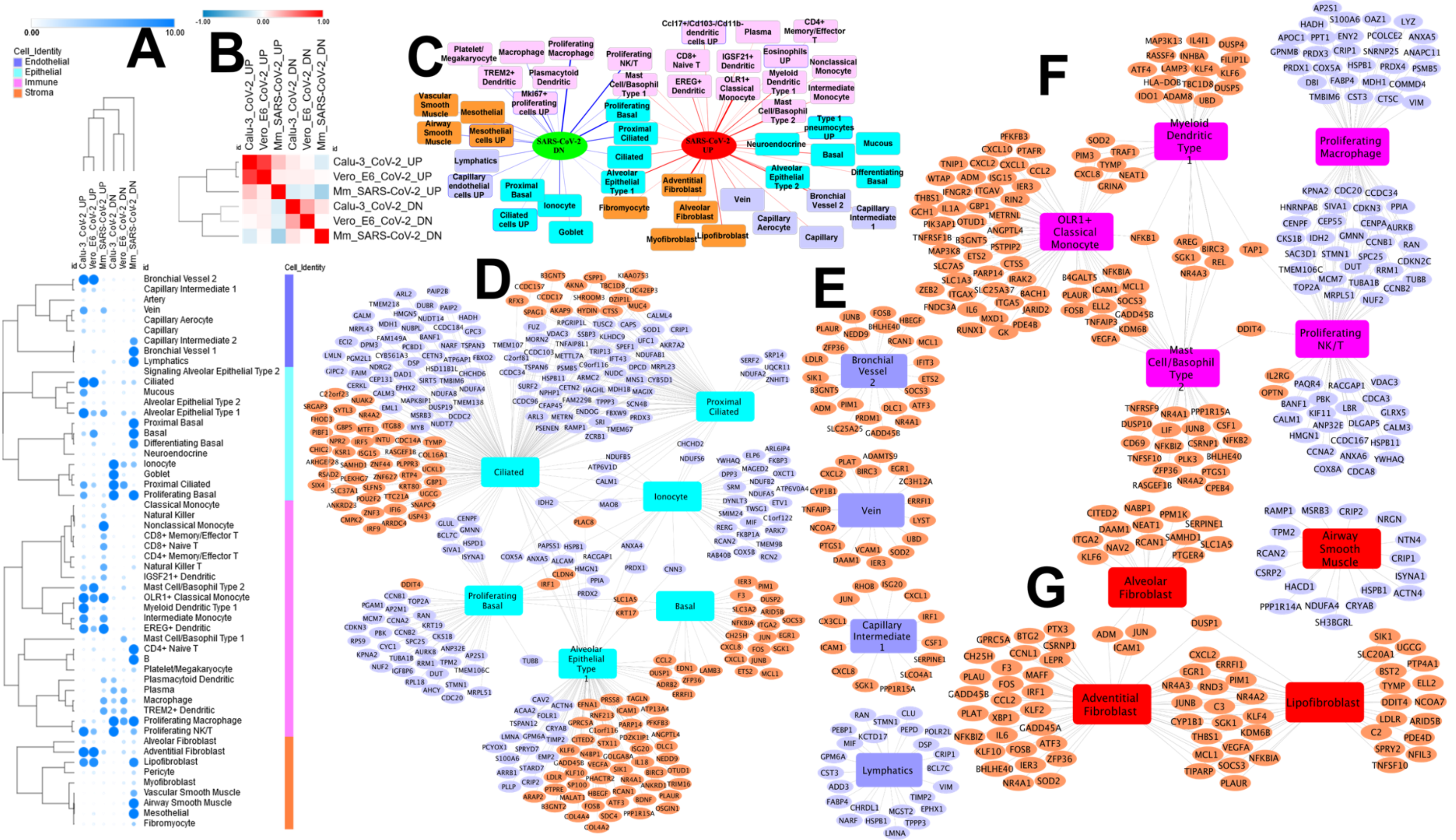

Figure S2: Lung cell type marker enrichment in consensus differentially expressed genes from the 3 SARS-CoV-2 infection models. **(A)** Enrichment heatmaps of single cell markers from normal human lung 22 among the differentially expressed genes from the 3 models of SARS-CoV-2 infection. The size and intensity of the colors in the circles is proportional to the significance of enrichment as measured by Fisher's exact test (negative log p-values). **(B)** Overlap heatmap of differentially expressed genes among the three SARS-CoV-2 infection models. **(C)** Network of enriched cell types from normal human lung 22 in the consensus differentially expressed genes from the 3 models of SARS-CoV-2 infection. The different colored rectangles are various cell types. The pink colored rectangles are myeloid cell types, purple-colored ones are lymphoid, green-colored ones are endothelial, blue-colored rectangles are epithelial, and the orange-colored nodes are stromal cell types. The width of the edges is proportional to the significance of the cell type enrichment (negative log p-value) measured by Fisher's exact test. **(D)-(G)** Network representations of enriched cell types and associated consensus differentially expressed genes from the 3 SARS-CoV-2 infection models. Consensus upregulated genes are in orange while the downregulated genes are in purple. The different panels represent epithelial, myeloid, lymphoid, endothelial, and stromal cell type enrichment networks along with their associated genes.

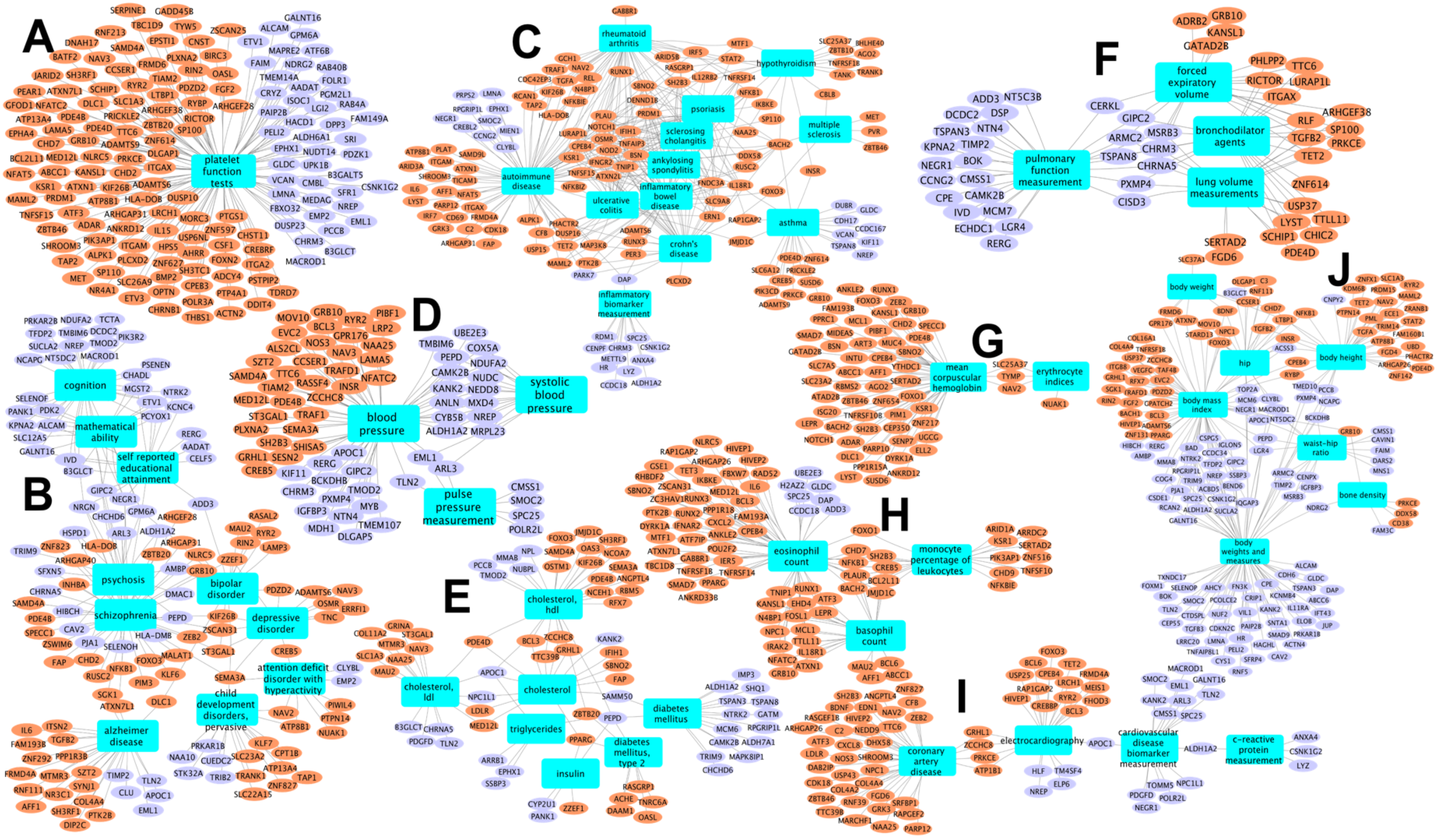

Figure S3: GWA loci enrichment in consensus differentially expressed genes from the 3 SARS-CoV-2 infection models. Network representation of select enriched PheGenI/GWA traits and their associated consensus DEGs from the 3 models of SARS-CoV-2 infection. Upregulated genes are in orange, downregulated genes are in purple, and the enriched traits are shown as blue colored rectangles. Networks are shown as different panels (A-J) based on their broad categorization, where possible. For instance, panel a shows immune system disorders, while panel g represents lung function measurements.

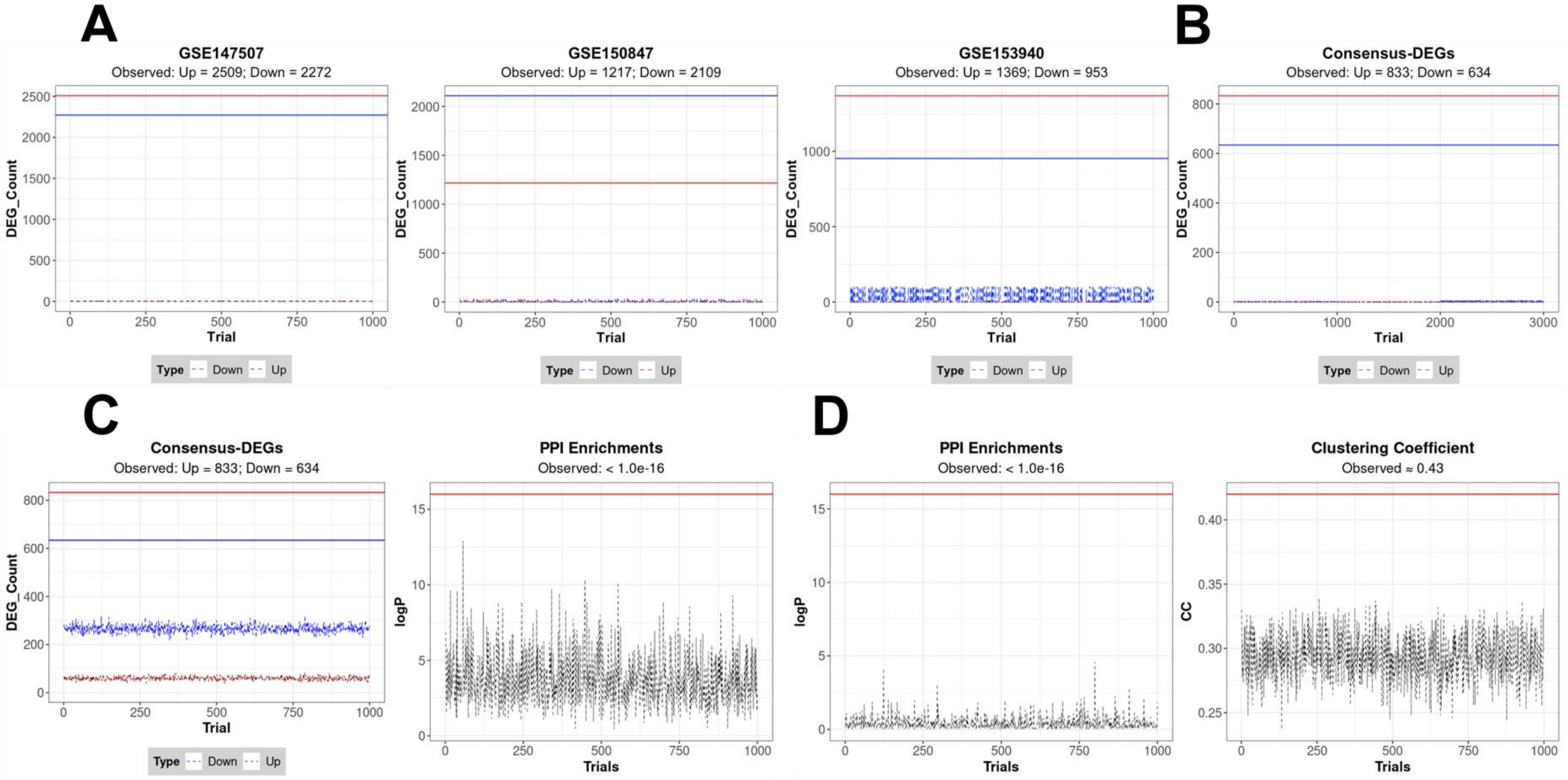

Figure S4: Robustness tests to validate the DEGs from the 3 SARS-CoV-2 infection models. **(A).** Plots of observed DEG counts in 1000 random trials where phenotype labels in each study were randomly permuted and DEGs were obtained based on the randomized labels. The actual counts of DEGs from each of the three SARS-CoV-2 infection models are indicated at the top of the plots below the GEO data set IDs. **(B).** Plots of consensus DEGs obtained by randomly permuting the phenotype labels from two of the three studies. This process was repeated 1000 times for each combination (3000 trials in total) and consensus DEGs were identified in each trial. **(C).** Plots of consensus DEG counts and PPI enrichments (negative log p-values) in randomized experiments where the DEGs were picked randomly from each model and used to identify the consensus candidates. The DEGs were then combined with the SARS-CoV-2 human interactants (336 genes) and tested for PPI enrichments. These two steps were repeated 1000 different times and the final consensus DEG counts (left) and PPI enrichment p-values (right) were recorded in each step. Actual DEG counts (upregulated = red; downregulated = blue) and the observed PPI significance level (< 1.0e-16) are represented as horizontal lines within each plot **(D).** Final set of randomized experiments where both the consensus DEGs (1467 genes) and the SARS-CoV-2 virus-host interactants (336 genes) are randomly generated in each trial. In addition to the PPI enrichment p-values (left), we also retrieved and plotted the average clustering coefficient values (right) in each trial.
