# Supplementary material for: Secondary analysis of transcriptomes of SARS-CoV-2 infection models to characterize COVID-19
Source: Patterns (N Y). 2021 Apr 5;2(5):100247. doi: 10.1016/j.patter.2021.100247 (PMC8020120; doi:10.1016/j.patter.2021.100247)
Supplement: Document S2. Article plus supplemental information [file mmc17.pdf]

Patterns

Article

Secondary analysis of transcriptomes of SARS-CoV-2 infection models to characterize COVID-19

Graphical abstract

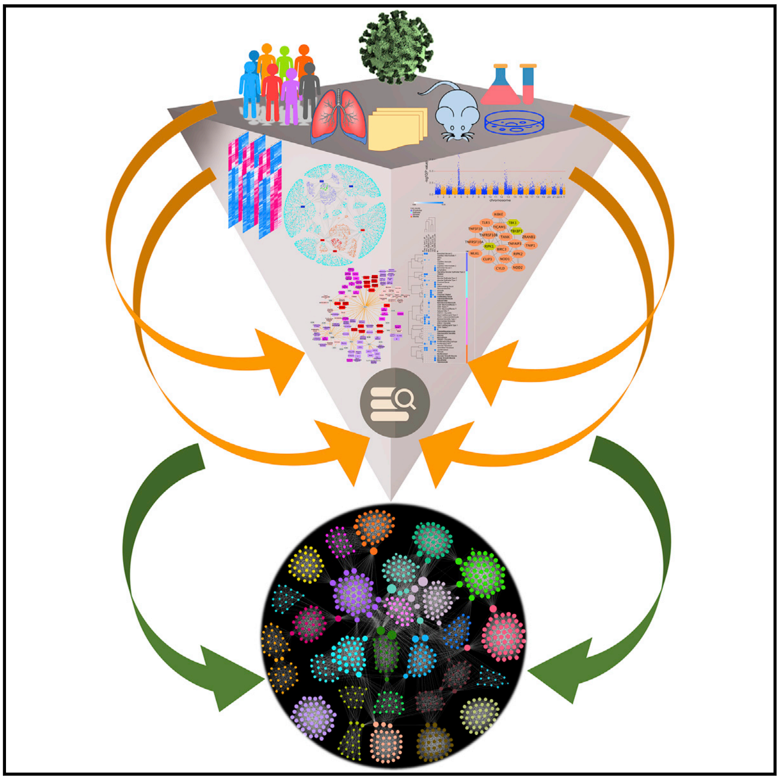

Authors

Sudhir Ghandikota, Mihika Sharma, Anil G. Jegga

Correspondence

anil.jegga@cchmc.org

In brief

We report a data-driven, network-based workflow to identify gene and functional modules in COVID-19 through joint analysis of gene expression data from three model systems of SARS-CoV-2 infection. Bringing together a consensus gene expression signature from these model systems and analyzing it jointly with other omics data, we build clusters of higher-order multifeature machines that provide a basis for addressing several basic and translational research questions and generation of hypotheses.

Highlights

- Defined a consensus gene signature across three models of SARS-CoV-2 infection
- Characterized subnetworks of host proteins interacting with SARS-CoV-2 proteome
- Integrated a wide range of COVID-19 and related data to build functional modules
- Identified gene functional modules that can further the understanding of COVID-19

Ghandikota et al., 2021, Patterns 2, 100247
May 14, 2021 © 2021 The Author(s).
https://doi.org/10.1016/j.patter.2021.100247

Patterns

CellPress
OPEN ACCESS

Article

Secondary analysis of transcriptomes of SARS-CoV-2 infection models to characterize COVID-19

Sudhir Ghandikota,[1,2] Mihika Sharma,[1] and Anil G. Jegga[1,2,3,4,*]
[1]Division of Biomedical Informatics, Cincinnati Children's Hospital Medical Center, 240 Albert Sabin Way, MLC 7024, Cincinnati, OH 45229, USA
[2]Department of Computer Science, University of Cincinnati College of Engineering, Cincinnati, OH 45221, USA
[3]Department of Pediatrics, University of Cincinnati College of Medicine, Cincinnati, OH 45267, USA
[4]Lead contact
*Correspondence: anil.jegga@cchmc.org
https://doi.org/10.1016/j.patter.2021.100247

THE BIGGER PICTURE This study is based on the premise that combining information from multiple layers of data can result in new biologically interpretable associations in several ways. The underlying and unifying theme of this study is data integration, data mining, and meta-analysis for pattern detection that supports knowledge discovery and generation of hypotheses. The methods and the workflow used are disease agnostic and can be applied to any disease or phenotype that has multiple models and heterogeneous data elements. By integrating and joint analysis of several heterogeneous data types (multiple disease models, viral-host protein interaction data, single-cell RNA-sequencing data, protein-protein interactions, and genome-wide association study data), gene functional modules are identified that can have direct bearing on furthering the understanding of COVID-19.

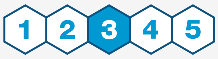

Development/Pre-production: Data science output has been rolled out/validated across multiple domains/problems

SUMMARY

Standard transcriptomic analyses alone have limited power in capturing the molecular mechanisms driving disease pathophysiology and outcomes. To overcome this, unsupervised network analyses are used to identify clusters of genes that can be associated with distinct molecular mechanisms and outcomes for a disease. In this study, we developed an integrated network analysis framework that integrates transcriptional signatures from multiple model systems with protein-protein interaction data to find gene modules. Through a meta-analysis of different enriched features from these gene modules, we extract communities of highly interconnected features. These clusters of higher-order features, working as a multifeatured machine, enable collective assessment of their contribution for disease or phenotype characterization. We show the utility of this workflow using transcriptomics data from three different models of SARS-CoV-2 infection and identify several pathways and biological processes that could enable understanding or hypothesizing molecular signatures inducing pathophysiological changes, risks, or sequelae of COVID-19.

INTRODUCTION

In vitro and *in vivo* disease models often fail to completely recapitulate the disease manifestations in humans. Integrated secondary analysis approaches that can identify disease-related gene modules by leveraging knowledge from multiple disease models can find physiological functions in a disease. Functional complexes that arise out of these gene or protein modules are known to represent distinct biological functions.[1,2] Similarly, feature networks comprising biological processes, pathways, phenotypes, and cell types represent a higher-order multifeatured machines collectively working toward a common goal. Based on this premise, we implemented a multilayered data-mining methodology that leverages protein modules to build functional modules or complexes in a disease. These functional complexes are built by linking together several heterogeneous data types such as single-cell RNA-sequencing (RNA-seq) markers, protein-protein interactions, and phenotype-genotype

This is an open access article under the CC BY license (http://creativecommons.org/licenses/by/4.0/).

associations. To demonstrate the utility of this joint analysis approach, we analyzed transcriptomic data from two *in vitro* models (Calu-3 and Vero E6 cells) and one *in vivo* model (Ad5-hACE2-sensitized mice) of SARS-CoV-2 infection.

Coronavirus disease 2019 (COVID-19), caused by SARS-CoV-2, has affected more than 75 million people with more than 1.6 million deaths worldwide including ~17.3 million confirmed infections and >311,000 deaths in the United States (World Health Organization, December 20, 2020). The limited and emerging stages of data and information surrounding this disease, and the necessity to find effective interventions (e.g., vaccines, small molecules), provides a strong rationale for a multilayered, secondary analysis of existing data collected from different models and studies. Some of the noteworthy discoveries surrounding SARS-CoV-2 are direct offshoots of secondary data analysis using available omics data generated in pre-COVID-19 times. These existing data include single-cell RNA-seq (scRNA-seq) data[3,4] from the Human Cell Atlas consortium or eQTL variant data[5] from the Genotype Tissue Expression (GTEx) database.[6] Thus, leveraging the available repository of datasets and information, even if they were not designed specifically to study COVID-19, can provide a jump start to discover different sides of this disease. Recently there have been several studies reporting network analysis-based approaches applied to both COVID-19- and non-COVID-19-related data to detect tissue-specific[7,8] or pan-tissue[9] networks of interacting genes specific to SARS-CoV-2 infections. These studies differ in the input "seed" genes used to construct the networks; some studies are focused on the SARS-CoV-2 entry-associated receptors and/or proteases[7,9] while the others use an expanded set of virus-host interactants in SARS-CoV-2.[8,10] However, most of these methods do not consider the differentially expressed genes (DEGs) in the host following the SARS-CoV-2 infection in their analysis. A recently published study[11] used differentially expressed host genes in SARS-CoV-2-infected bronchial epithelial cells (NHBE) along with the SARS-CoV-2 entry receptor ACE2 and SARS-CoV-2 entry-associated protease TMPRSS2 to construct a host gene regulatory network. This study, however, is based on a single SARS-CoV-2 infection model with a limited set (three samples) of SARS-CoV-2 infection samples. Additionally, the study did not consider other host-virus interactants specific to SARS-CoV-2 virus. To overcome these limitations and address some of these issues, we used transcriptomic data from three model systems (two *in vitro* and one *in vivo*) of SARS-CoV-2 infection, SARS-CoV-2 viral-host protein interaction data, and analyzed them jointly with non-COVID-19/SARS-CoV-2 data. For the latter, we used the scRNA-seq markers from three human lung studies, protein-protein interactions, and genome-wide association study (GWAS) data (Figure 1). While we acknowledge the complexity of SARS-CoV-2 infection, we believe that our study supports knowledge discovery and formulation of testable hypotheses for COVID-19 pathogenesis.

RESULTS

Consensus transcriptome in SARS-CoV-2 infection

The pathophysiology of most viral infections is associated with host protein complexes, which are manipulated to hijack the individual cell biological processes. Therefore, to evaluate this phenomenon, we first built an interactome around the consensus transcriptome of SARS-CoV-2 infection. To obtain a consensus transcriptomic signature, we considered DEGs in at least two of the three SARS-CoV-2 models[12–14] compared (i.e., two cell lines, namely, transformed lung-derived Calu-3 cells and VeroE6 cells, and a mouse model) (Figure 2A and Table 1). A strong concordance was seen among the upregulated and downregulated gene signatures from the three models. A total of 732 DEGs (537 upregulated and 195 downregulated) were shared between the SARS-CoV-2-infected human Calu-3 and non-human primate VeroE6 cell lines (Figure 2B). Similarly, we found 325 upregulated and 369 downregulated genes common between the Calu-3 model and Ad5-hACE2-sensitized mice. While there was an overall concordance among the DEGs, each of the three models also had several DEGs unique to them (Figure 2C and Table S1). We further validated these DEGs by comparing them with a transcriptomic signature from COVID-19 patients (GEO: GSE152075; nasopharyngeal swabs from 430 patients and 54 controls).[15] There was a stronger concordance with the transcriptomic signature from the Calu-3 and the Ad5-hACE2-sensitized mouse model systems than the one from the VeroE6 cell line model (Figure S1). Finally, a total of 1,467 consensus genes (833 upregulated and 634 downregulated) were found (Figure 2C and Table S2) from the three disease models. This included 106 genes upregulated and 41 genes downregulated in all three model systems (Figures 2C and 2D), representing the "core" dysregulated transcriptome in SARS-CoV-2 infection. Both these sets of consensus signatures were enriched for several functional terms (Tables S3, S4, and S5) and human lung cell-type markers (Table S6 and Figure S2). Additionally, these gene sets were also enriched for several physiological and pathological traits (from the Phenotype-Genotype Integrator [PheGenI][16] and GWAS catalog[17] databases) (Tables S7 and S8; Figure S3).

Interactome of consensus transcriptome of SARS-CoV-2 infection and virus-host protein-protein interactions

To build a consensus SARS-CoV-2 interactome, we used the SARS-CoV-2-human virus-host protein-protein interaction (PPI) dataset comprising 332 human proteins involved in assembly and trafficking of RNA.[18] These are in addition to the SARS-CoV-2 entry receptor ACE2, and SARS-CoV-2 entry-associated proteases, namely, TMPRSS2, CTSB, and CTSL. More than half (151 genes) of these 336 SARS-CoV-2-human interacting proteins were differentially expressed in at least one of the three model systems (Figure 3A). Of these, 29 genes (16 upregulated and 13 downregulated) were part of the consensus signature.

Using the disease consensus transcriptomic signature and the SARS-CoV-2-proteome interacting human proteins as an input, we queried the STRING (v11) database[19] and generated a DEG-PPI integrated network. Only the interactions with highest confidence score (0.9) or experimental interaction score of 0.7 or more in STRING were used. We observed an enrichment for PPIs ($p < 1.0 \times 10^{-16}$) among the combined gene set (Figure 3B). In other words, this combined set of SARS-CoV-2 consensus signature and SARS-CoV-2-human interaction map have significantly more interactions among themselves than would be expected for a random set of proteins of similar

CellPress
OPEN ACCESS

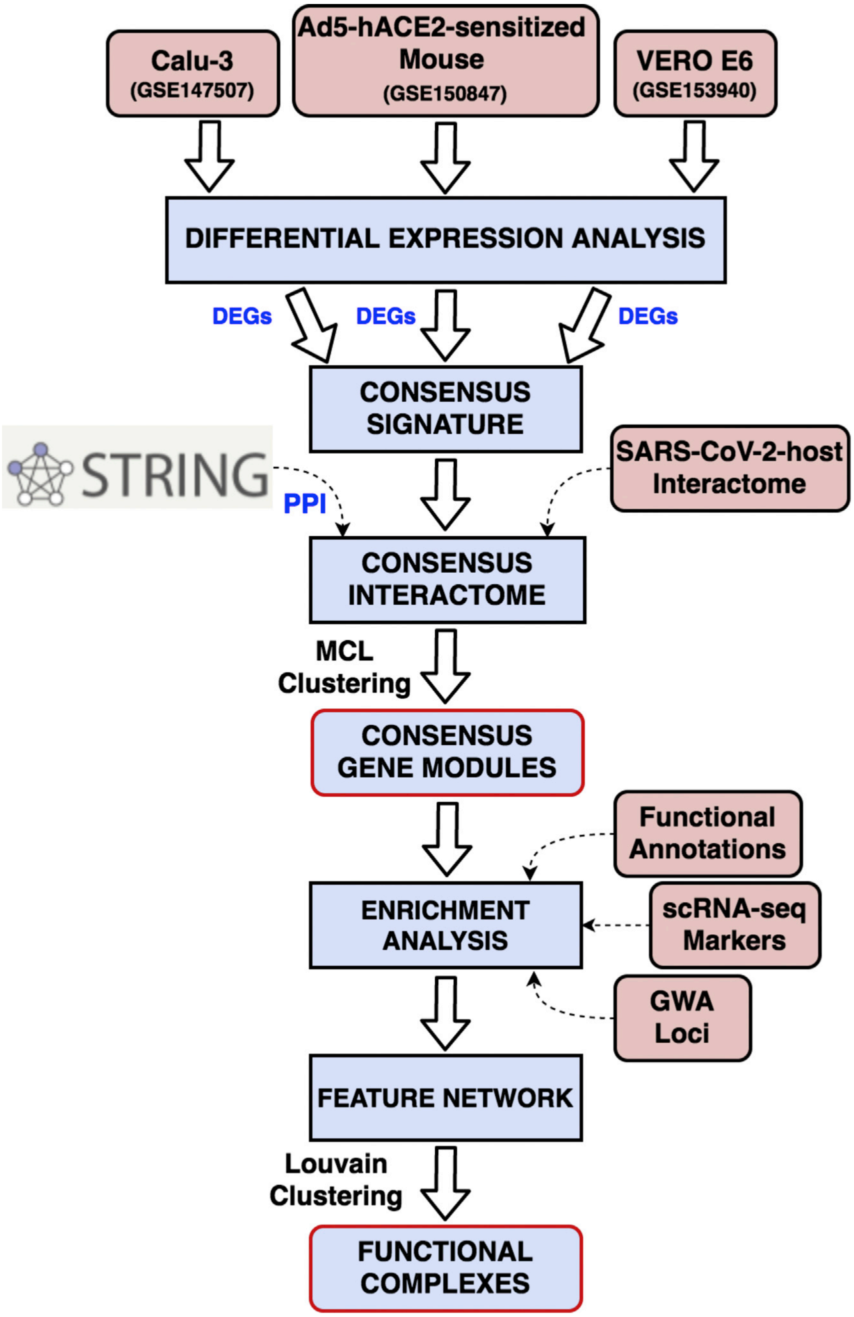

Figure 1. Schematic representation of the workflow

Transcriptomic data from three SARS-CoV-2 infection models are processed to identify differentially expressed genes (DEGs). Genes that are up- or downregulated in two out of three models are considered as "consensus signature." The consensus DEGs, along with the SARS-CoV-2-human virus-host interactome, are used to build an integrated network based on known protein-protein interactions (PPIs) retrieved from STRING database (v11). A Markov clustering (MCL) algorithm is then used to identify modules of highly interconnected genes from this integrated interactome. These gene modules are characterized through functional enrichments (pathways, biological processes), lung single-cell markers, and phenotypic trait (genome-wide association [GWA]) loci. In the final step, an enriched feature network is constructed using all the enriched terms and features from the modules to extract functional complexes or communities of highly interconnected features.

size drawn from the genome. We next identified network clusters from this joint interactome using a Markov clustering (MCL) algorithm. In brief, MCL clusters a network to determine modules of genes with more intramodular (within the module) than intermodular (with other modules) interactions. Each gene can only be assigned to a single module through this method. The inflation factor parameter determines the granularity (or "tightness") of the clusters and thereby the cluster size. In all our experiments with SARS-CoV-2 infection models, we used the default inflation parameter (2.5). With MCL clustering, we found 153 clusters of varying gene counts (Table S9). We selected 35 candidate clusters with each having at least five genes. These 35 clusters were made up of a total of 797 genes of which 627 were consensus DEGs in SARS-CoV-2 infection models (see Figures 3C–3H for six example clusters and Table S9 for more details). Of the 35 clusters, 29 clusters had at least one gene-encoding protein that interacts with the SARS-CoV-2 proteome. We hypothesize that these SARS-CoV-2-targeted human protein clusters are informative in deciphering the COVID-19 pathophysiology and inferring the function of the SARS-CoV-2 targets based on other members in the protein clusters.

Characterization of SARS-CoV-2-targeted human protein modules

Gene clusters: Functional enrichment

The next step in our multilayered approach was to obtain enriched biological processes and pathways for the identified gene modules (Table S10). Cluster C-1 (190 genes) was enriched for innate immune response (48 genes) and type I interferon signaling (26 genes) while genes from cluster C-2 (92 genes) were involved in transport regulation (31 genes) and tube development (31 genes). We also found genes associated with abnormal cardiovascular development (21 genes) in cluster C-2. Clusters C-7 (20 genes) and C-8 (20 genes) had genes associated with abnormal interleukin and cytokine secretion phenotypes. Clusters C-12 (14 genes), C-28 (6 genes), and C-23 (8 genes) were all enriched for mitochondrion translation, organization, and transport. Finally, several genes regulating circadian rhythm in mammals (*NFIL3*, *PER1*, *PER2*, *PER3*, and *SIK1*) were seen in cluster C-25 (7 genes).

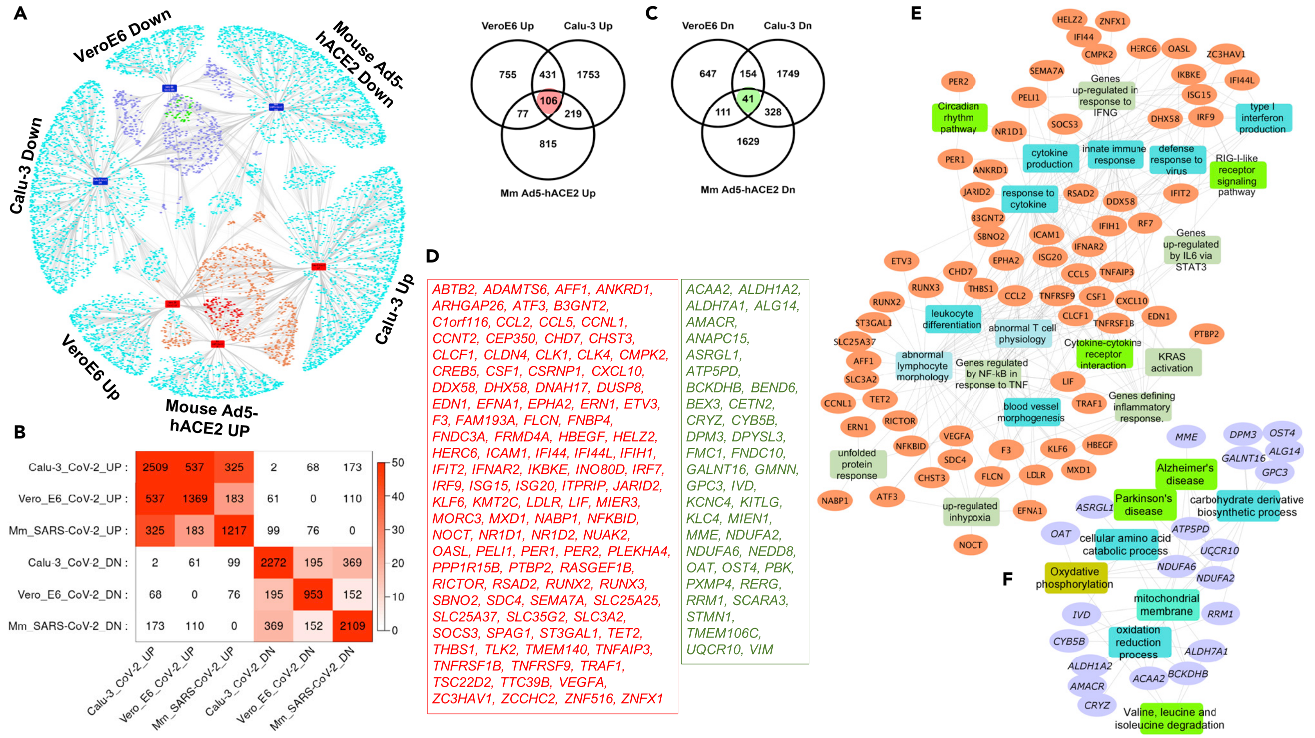

Figure 2. Transcriptomic overlaps among the two *in vitro* and one *in vivo* SARS-CoV-2 infection models

(A) Network of DEGs from the three SARS-CoV-2 infection models (Calu-3, VeroE6, and Ad5-hACE2 mice). The orange- and navy-blue-colored nodes are genes upregulated or downregulated, respectively, in at least two models. The red- and green-colored nodes are genes that are up- or downregulated in all the models compared.

(B) Heatmap indicating the transcriptomic overlaps between the different SARS-CoV-2 infection models. The size and significance of the overlaps is measured using the gene counts and Fisher's exact test, respectively.

(C) Venn diagram showing comparison of the up- or downregulated DEGs in the three models.

(D) List of the "core" upregulated (106 genes, red font) and downregulated (41 genes, green font) genes in all the three model systems.

(E and F) Network representation of enriched biological processes and pathways in the core upregulated (E) or downregulated (F) genes from the SARS-CoV-2 infection models. Orange- and purple-colored nodes are genes up- or downregulated, respectively. The different colored rectangles are enriched biological processes and pathways. Enrichment analysis was done using the ToppFun application of the ToppGene Suite, and network was generated using the Cytoscape application.

Gene clusters: Lung single-cell markers

We next evaluated the candidate gene modules for lung single-cell associations by performing enrichment analysis of the modules against single-cell marker gene sets compiled from three different human lung scRNA-seq studies.[20–22] Of the 35 selected gene clusters, 17 clusters (633 genes) were enriched for markers of at least one lung cell type (Figure 4 and Table 2). Cluster C-1 (190 genes) was enriched for proliferating cells including proliferating epithelial (e.g., proliferating basal), lymphoid (e.g., proliferating T cells, proliferating natural killer cells), and myeloid (e.g., proliferating macrophages) cell types. Cluster C-2 (92 genes) was heterogeneous showing enrichment for epithelial, mesenchyme, vascular endothelial, lymphoid, and myeloid cell types. Cluster C-9 (18 genes) showed enrichment for fibroblasts, myofibroblasts, and smooth muscle cells and shared enrichments with clusters C-1, C-2, and C-3. Some of the clusters were found to be specifically enriched for certain cell types. Ionocyte cell marker[22] genes, for instance, were specific to cluster C-5 (40 genes; 12 markers); clusters C-7, C-11, and C-13 were specifically enriched for myeloid cell markers (Table S11).

Gene clusters: Genotype-phenotype associations

The 35 genes clusters also showed enrichment for several physiological and phenotypic traits that provide insights into COVID-19 pathogenesis (Tables S12 and S13). Among the most significantly enriched traits were respiratory system disease (clusters C-7 and C-8), asthma (C-7), autoimmune disease (clusters C-7 and C-29), allergic rhinitis (C-7), immune system disease (cluster C-7 and C-8), and diabetes (C-15). We also observed risk genes associated with several inflammatory disorders such as inflammatory bowel disease and Crohn's disease (C-7), ulcerative colitis (C-8), rheumatoid arthritis (clusters C-7 and C-8), and ankylosing spondylitis (C-8). Apart from elucidating the pathophysiology of COVID-19, the enriched traits can potentially help the researchers to understand or formulate hypotheses surrounding the long-hauler patients or survivors. For instance, could COVID-19 be a risk factor for autoimmune or neurodegenerative disease? A plausible mechanism could be through an overactivated innate immune system.[23–25] Both acute and delayed neurological and neuropsychiatric effects have been associated with previous viral pandemics.[26,27]

Table 1. List of differentially expressed genes (0.6 logFC; FDR p $\leq$ 0.05) from the three SARS-CoV-2 infection models

Differentially expressed gene list name	No. of DEGs	GEO ID	Reference
Calu3 SARS-CoV-2: downregulated	2,272	GSE147507	Blanco-Melo et al.[12]
Calu3 SARS-CoV-2: upregulated	2,509		
Ad5-hACE2-sensitized mouse SARS-CoV-2: downregulated	2,109	GSE150847	Riva et al.[13]
Ad5-hACE2-sensitized mouse SARS-CoV-2: upregulated	1,217		
Vero E6 SARS-CoV-2: downregulated	953	GSE153940	Sun et al.[14]
Vero E6 SARS-CoV-2: upregulated	1,369		
Overall number of unique SARS-CoV-2 DEGs: 8,286			

SARS-CoV-1-targeted human protein modules

To demonstrate that the proposed workflow is disease agnostic and to identify modules that are specific to SARS-CoV-2 infection, we implemented the same workflow for another corona virus disease caused by SARS-CoV-1. To do this, we first extracted DEGs from three different SARS-CoV-1 infection models[28–30] (Calu3 model and two mouse models), and generated the consensus DEGs. There were 699 upregulated and 1,385 downregulated genes that were differentially expressed in at least two out of the three model systems. To generate the SARS-CoV-1-targeted human protein modules, we used 366 host-SARS-CoV-1 protein interactions identified on the basis of localization of viral proteins in human cells.[31] Comparing the DEGs and virus-host protein interactions of SARS-CoV-1 and SARS-CoV-2, we found over 300 DEGs (196 upregulated and 119 downregulated) and 135 viral interactants shared, and a large number of DEGs and protein interactions unique to each of them. We next generated SARS-CoV-1-targeted human protein modules following the same steps as described previously for SARS-CoV-2. We identified 68 modules that had at least five genes (Table S14). We also computed functional and lung cell marker enrichments for the SARS-CoV-1 modules. By analyzing the module compositions from both of the analyses (SARS-CoV-2 and SARS-CoV-1), we identified candidate modules that are potentially unique to each of these viruses. For instance, cluster C-5 (40 genes) from the SARS-CoV-2 interactome contained more than 90% of its gene members (37 out of 40) from the SARS-CoV-2 consensus signature or protein interactions. Interestingly, this module was enriched for marker genes from ionocytes and proximal ciliated cells, and several neurodegenerative disease pathways. Similarly, 9 out of 11 genes in cluster C-15 were specific to the SARS-CoV-2 interactome, which included genes belonging to trans-synaptic signaling and neurotrophic factor-mediated Trk receptor signaling pathways. Among lung cell markers, proliferating epithelial and basal cells along with transitional AT2 cell markers were specifically enriched in our identified SARS-CoV-2 protein modules. Likewise, we observed multiple functional pathways (e.g., TRAIL [tumor necrosis factor-related apoptosis-inducing ligand] signaling and IL12 [interleukin-12]-mediated signaling pathways), biological processes (e.g., endoderm formation, response to oxygen radical), and phenotypes (e.g., arteriosclerosis, abnormal mitochondrial crista morphology) enriched specifically in SARS-CoV-2. We also identified few protein modules containing a significant number of genes associated with both infections, potentially representing the pan-viral disease mechanisms involved (Table S14).

Meta-analysis of candidate gene modules and enrichment network visualization

To identify the semantic concordance between the enriched cell types, phenotypic traits, and functional terms for different gene clusters, we next undertook meta-analysis across all the enrichments. We selected a subset of enriched terms (top ten enriched terms from Gene Ontology—Biological Process, Reactome pathways, mouse phenotype), cell types, and traits (both PheGenI and GWAS catalog) from each of the 35 candidate clusters and converted them into a network layout. We used Gephi (https://gephi.org), an open-source graph visualization platform,[32] to construct and visualize the functional network. In this dense enriched feature network (1,198 nodes and 31,065 edges, Table S15), the enriched terms (biological processes, pathways, phenotypic traits, cell types) are represented as nodes, and two nodes are connected if they share at least one or more of the 35 candidate gene clusters from the combined interactome map. Since subunits of a functional complex (a cluster of, e.g., pathways, cell types, biological processes, phenotype) work toward the same biological goal, prediction of an unknown pathway or biological process or a phenotype as part of this complex also allows increased confidence in the annotation of that functional cluster. Additionally, by doing this, potential redundancies across different sources (e.g., ontology or cell types) could be reduced, apart from enabling interpretation of the enrichment results through intracluster and intercluster similarities of enriched terms.[33] We therefore investigated the substructure of the feature network by estimating community membership modules using the Louvain algorithm[34] (implemented in Gephi). Louvain clustering is a fast, iterative algorithm that is based on optimizing the modularity score[35] and is computationally fast, efficient, and suitable for large modular networks. The resolution parameter can be used to maintain the balance between module count and the individual cluster tightness. A low-resolution parameter value would lead to smaller, more tightly connected clusters and vice versa. With a resolution set to 0.25, we found 31 communities of highly interconnected biological terms and a high modularity score of 0.672 (Figure 5). Visualizing these functional complexes, we observed high concordance between the functional terms, cell-type marker, and phenotype enrichments among the candidate gene modules (Table 3). For instance, cluster C-10 was enriched for vascular

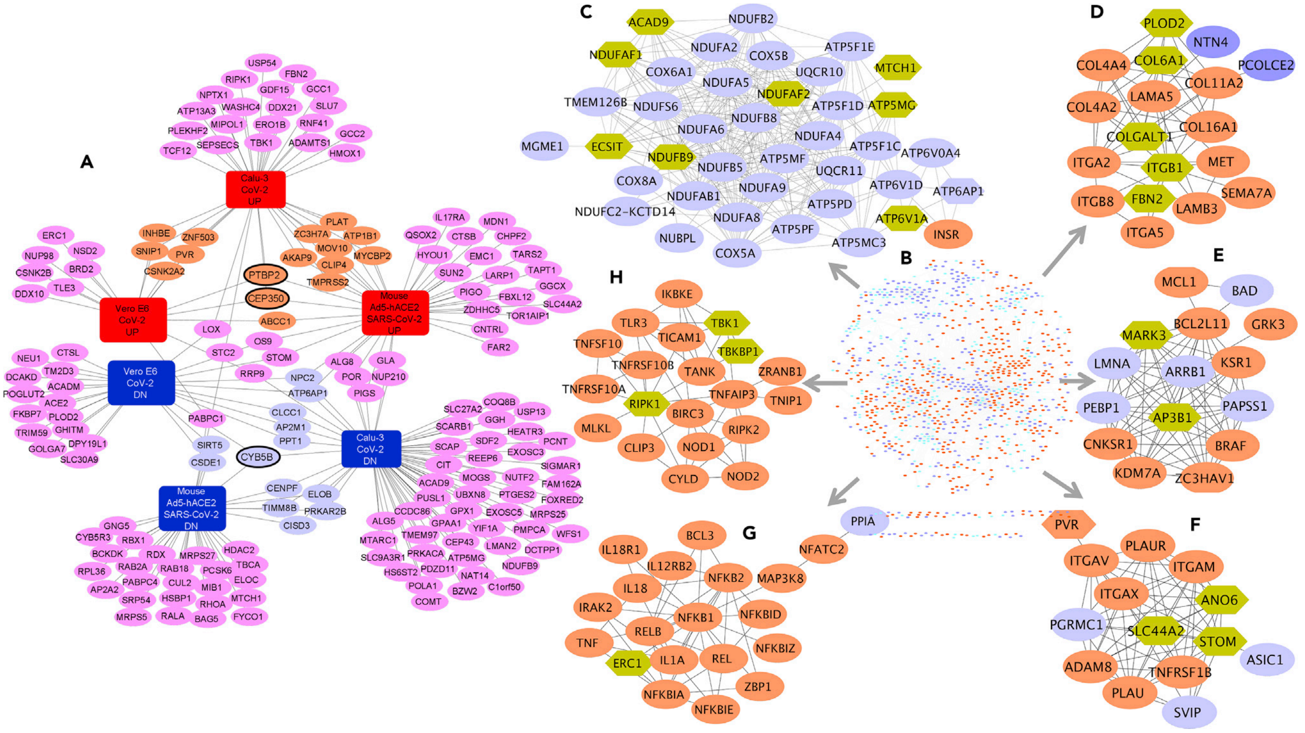

Figure 3. Network of consensus DEGs of SARS-CoV-2 infection models and SARS-CoV-2-host protein-protein interactions

(A) Network of 176 DEGs of three SARS-CoV-2 infection models that encode proteins interacting with SARS-CoV-2 viral proteins. The orange- and purple-colored nodes are consensus up- or downregulated genes, respectively, while the three genes with black border (*PTBP2*, *CEP350*, and *CYB5B*) are part of the core set of genes.

(B) STRING-based interaction network of consensus DEGs and SARS-CoV-2-human viral-host protein interactome.

(C–I) Example gene clusters from the consensus DEG and SARS-CoV-2-host integrated interactome. Clusters (based on MCL network clustering) shown are C-5 (C), C-9 (D), C-11 (E), C-13 (F), C-7 (G), and C-8 (H). Consensus up- and downregulated genes are in orange and purple, respectively, while the hexagonal genes are part of the SARS-CoV-2-host protein interactome, directly interacting with the consensus DEGs.

endothelial and smooth muscle cells, platelet degranulation, extracellular matrix, cell-substrate adhesion, and FOXP3 targets (Figure 5). Elucidating the role of platelets in the thrombotic complications of COVID-19, two recent studies[36,37] reported that platelet hyperactivity contributes to the COVID-19-related coagulopathy. Furthermore, endothelial cell dysfunction and impaired microcirculatory function are reported to contribute to COVID-19 severity including venous thromboembolic disease and multiple organ involvement.[38] Foxp3 is a master regulator of regulatory T (Treg) cells, and its expression is associated with the immunosuppressive activity of these cells. Deficiency of functional Treg cells caused by mutations of Foxp3 leads to spontaneous systemic multiorgan autoinflammatory phenotypes in mice.[39–42] Interestingly, $CD4^+CD25^+FoxP3^+$ regulatory T cell-based therapies are proposed for COVID-19 patient management.[43] Similarly, clusters C-11 and C-13, and C-7 (Figures 3E–3G) were enriched for Toll-like receptor signaling pathway, cytokine-cytokine receptor interaction, nuclear factor κB (NF-κB) signaling, CD40 signaling, and myeloid cell types (conventional dendritic cells, mast cells, and monocytes). These clusters showed enrichment for abnormal interleukin secretion and T cell physiology and for several GWA loci such as granulocyte count, inflammatory biomarker measurement, Crohn's disease, and ulcerative colitis (Figure 5 and Table 3).

DISCUSSION

We report a data-driven, network-based workflow to identify gene and functional modules in a disease through joint analysis of disease-specific and non-disease-specific data elements. By integrating high-confidence protein-protein interactions with disease-specific transcriptomic signatures, we first identified protein modules that could represent perturbed states in disease. As a first pass of characterizing these modules, we leverage existing heterogeneous omics data including different biological processes, pathways, single-cell associations, and genetic traits. Next, we construct a feature network using the enriched terms from different perturbed modules. These higher-order multifeature machines, or functional modules overlaid on protein modules representing perturbed states, enable us to identify biologically interpretable mechanisms underlying disease pathophysiology. This approach is disease agnostic and can be applied to any disease or phenotype that has one or more model systems with transcriptomic data.

We demonstrate the utility of our approach by undertaking a secondary analysis of transcriptomic data from three models of SARS-CoV-2 infection. By integrating and analyzing the transcriptomic data from COVID-19 *in vitro* and *in vivo* models in the context of SARS-CoV-2-human virus-host protein interaction

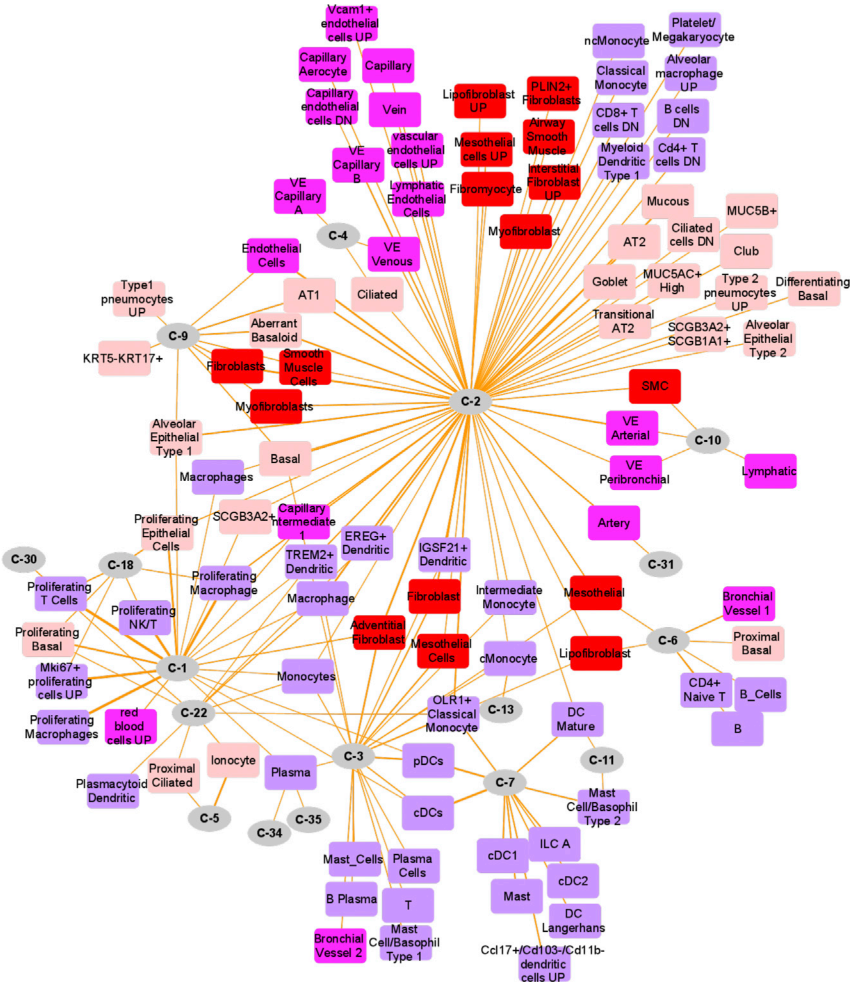

Figure 4. Lung single-cell marker enrichments in gene clusters from the integrated interactome of consensus DEGs of SARS-CoV-2 infection models and SARS-CoV-2-host protein-protein interactions

Marker enrichment network of 17 candidate clusters (circular nodes) that were enriched for at least one lung cell type (rectangular nodes) marker in human lung.

map, single-cell signatures of lung, gene annotations, and human genotype-phenotype associations, we have identified several functional modules that can have direct bearing on furthering the understanding of this devastating pandemic. We also demonstrate the disease-agnostic nature of our approach through analysis of transcriptomic data from infection model systems of another corona virus (SARS-CoV-1). Furthermore, we were able to identify SARS-CoV-2-specific gene modules and unique functional mechanisms by comparing the results from the SARS-CoV-2 with that of SARS-CoV-1 infection model systems. The various categories of cellular functions and phenotypic traits found by meta-analyses of SARS-CoV-2 model systems recovered both expected and potentially novel biological insights of COVID-19. The gene-level and higher-order feature-level clusters emerging from the joint analysis of COVID-19- and non-COVID-19 related data can serve as valuable resources for the scientific community to formulate or further investigate hypotheses.

Limitations of the study

Our methodology holds certain limitations. The composition of the protein modules is dependent on the transcriptomic signature used, and any heterogeneity in the transcriptomic data can affect the module compositions. For instance, lack of transcriptomic concordance between different disease model systems could result in gene or protein modules that are very diverse. Although the Ad5-hACE2-transduced mice develop pneumonia after infection with SARS-CoV-2 and are useful for evaluation of vaccines and antiviral therapies, the infection is non-lethal.[14,44,45] A comparison of the transcriptomic signatures from the three model systems with DEGs from human samples indeed showed several genes that are uniquely dysregulated in COVID-19 patients (GEO: GSE152075)[15] suggesting the inherent drawbacks of current *in vitro* and *in vivo* models of COVID-19. Newer transcriptomic signatures as and when available from emerging refined and more representative *in vitro* and *in vivo* models of human COVID-19[46–49] can be leveraged and analyzed using the current workflow. The small sample size from the three disease models used in the study is another limitation. However, we performed multiple randomized trials to demonstrate the robustness of the three transcriptomic datasets used in the study (see experimental procedures and Figure S4). Nevertheless, further (*in vitro* and *in vivo*) validations are warranted to test the hypotheses arising from the current study. Recently there have been several studies and databases reporting COVID-19-specific databases,[33,50–52] which can be either used to compile additional consensus gene sets or for further functional characterization of the modules discovered in the current study.

The STRING-based PPI network data suffer from incompleteness and a certain degree of noise. There are no set standards for the optimal STRING interaction score cutoff. Furthermore, although Markov clustering is recommended for module detection,[53] there are no specific guidelines for inflation factor threshold nor for the functional annotation of modules. Nevertheless, to overcome some of these limitations, we used a very stringent cutoff score of 0.9 for STRING interactions and selected 2.5 (default) as the MCL inflation factor. The cluster composition can also vary depending on the clustering algorithm and parameters. Additionally, the choice of external (non-disease) data elements is likely dependent on the disease or phenotype being studied. For instance, to identify cancer driver modules, DNA-level alterations (e.g., variants, copy number alterations) and RNA-level regulation data have been proved to be more effective.[54] To

Table 2. Candidate clusters in SARS-CoV-2 DEG and interaction map along with their enriched lung cell types

Cluster	Enriched cell markers
C-1 (190 genes)	proliferating natural killer/T cells, proliferating basal, proliferating macrophage, adventitial fibroblasts, alveolar epithelial type 1
C-2 (91 genes)	proliferating epithelial, ciliated, proliferating macrophage, classical monocytes, alveolar epithelial type 1, adventitial fibroblasts
C-3 (81 genes)	adventitial fibroblasts, lipofibroblasts, bronchial vessel 2, classical monocytes, mast cells
C-4 (73 genes)	ciliated, capillary endothelial cells
C-5 (40 genes)	ionocytes, proximal ciliated
C-6 (34 genes)	bronchial vessel 1, lipofibroblasts, mesothelial
C-7 (20 genes)	dendritic cells, mast cells, classical monocytes
C-9 (18 genes)	alveolar epithelial type 1, fibroblasts, basal, myofibroblasts, smooth muscle cells
C-10 (17 genes)	lymphatic, peribronchial, arterial
C-11 (15 genes)	dendritic, mast cells
C-13 (14 genes)	classical monocytes
C-18 (10 genes)	proliferating epithelial, proliferating T cells
C-22 (8 genes)	ionocytes, macrophages, proliferating T cells
C-30 (6 genes)	proliferating T cells
C-31 (6 genes)	Arteries
C-34 (5 genes)	plasma cells
C-35 (5 genes)	plasma cells

Clusters with ≥ 5 genes enriched for at least one human lung cell type are shown. For a complete list of clusters and their enriched cell types see Table S9 and Table S11, respectively.

alleviate the issues related to noise and incompleteness in PPI networks, graph neural network implementations, which are robust to structural noise in input networks, could be useful. Adding the expression profiles as node features could also be an efficient way to introduce disease-specific transcriptomics data into the network-based analysis. Using attention-based implementations allows us to assign dynamic similarity weights to nodes (proteins) based on the similarity of their neighborhood-aggregated features. Additionally, we also plan to explore mechanisms that can integrate heterogeneous human transcriptomic data coming from distinct sources including nasopharyngeal swabs and peripheral blood mononuclear cells. In summary, bringing together a consensus gene signature from multiple disease model systems and analyzing it jointly with other omics data provide a basis for addressing several basic and translational research questions for existing and emerging diseases.

EXPERIMENTAL PROCEDURES

Resource availability

Lead contact

Further information and requests should be directed to and will be fulfilled by the lead contact, Anil G. Jegga (anil.jegga@cchmc.org).

Materials availability

This study did not generate any unique reagents.

Data and code availability

All data generated or analyzed during this study are included in this article and its supplemental information files. Also, the code for reproducing our result files and figures is accessible publicly at https://github.com/SudhirGhandikota/COVID19_secondary_analysis. Additional supplemental items are available Mendeley Data at https://doi.org/10.17632/3cwxv9swkc.1.

SARS-CoV-2 infection models: Differentially expressed genes

We used transcriptomic data from human (Calu-3) and non-human primate (VeroE6) cell lines, and from a mouse model (Ad5-hACE2) of SARS-CoV-2 infection (Table 1). The SARS-CoV-2 infection triggered transcriptome in Calu-3 cell lines (GSE147507)[12] is based on six samples with three each of mock treated or infected with SARS-CoV-2. The second transcriptome signature is based on mRNA profiles of control and 24-h post-SARS-CoV-2-infection (USA-WA1/2020, multiplicity of infection = 0.3) in Vero E6 cells (kidney epithelial cells extracted from an African green monkey (GEO: GSE153940).[13] The third dataset is from a mouse model using Ad5-hACE2-sensitized mice (GEO: GSE150847)[14] that develop pneumonia after infection with SARS-CoV-2, overcoming the natural resistance of mice to the infection. Raw data from GEO: GSE147507,[12] GSE153940,[13] and GSE150847[14] were obtained and analyzed using the Computational Suite for Bioinformatics and Biology (CSBB v3.0).[55] The raw data were downloaded from NCBI Sequence Read Archive (*ProcessPublicData* module), and the technical replicates were merged for individual samples before processing them (*Process-RNASeq_-SingleEnd* module). Quality checks[56] and quality trimming[57] were conducted prior to the transcript mapping/quantification step using the RSEM package.[58] Raw counts and transcripts per million were generated for all samples for further downstream analysis. Within each sample series, differential expression (DE) analysis was carried out based on treatment versus mock samples using the CSBB-Shiny server.[19] RUVSeq[59] was used to remove potential variation and sequencing effects from the data before performing DE analysis using edgeR.[60] DEGs were obtained by applying a 1.5-fold change threshold (i.e., $\log_2 FC \geq 0.6$ or $\log_2 FC \leq -0.6$) and a p value (false discovery rate [FDR] correction) of <0.05. For obtaining the human ortholog genes for mouse (*Mus musculus*) and green monkey (*Chlorocebus sabaeus*), we used ortholog mappings from the NCBI's HomoloGene.

SARS-CoV-2-human virus-host protein-protein interactions data

The SARS-CoV-2-human virus-host protein-protein interaction data included a set of 332 human proteins involved in assembly and trafficking of RNA viruses and shown recently through affinity purification and by mass spectrometry to interact physically with 26 of 29 SARS-CoV-2 structural proteins.[18] These are in addition to the SARS-CoV-2 entry receptor ACE2, and SARS-CoV-2 entry-associated proteases, namely, *TMPRSS2*, *CTSB*, and *CTSL*.

Consensus DEGs: Robustness tests

To test the robustness of DEGs and the consensus transcriptome from the three input disease models used in our framework, we performed four different randomized permutation tests. In the first set of experiments, we randomly permuted the phenotype labels in each individual study, identified the DEGs, and tried to obtain the consensus signature (genes that are differentially expressed in two or more studies). We repeated this for 1,000 iterations and observed that the number of DEGs found in each disease model is significantly less than the actual counts (Figure S4A). Consequently, we did not identify a consensus signature in any of the randomized trials due to the low DEG counts. Given the small sample sizes, the same phenotype combinations were repeated a few times in our trials. In the second set of experiments, we permutated the labels in two of the three studies and reused the original DEGs from the third study. We again repeated this process 1,000 times for each combination (3,000 random trials in total) and computed the consensus DEGs in each case. Here too we did not observe a significant number of consensus DEGs (Figure S4B) in any of our trials (<25 genes).

Our next set of experiments was designed to validate the level of connectivity observed among the SARS-CoV-2 consensus DEGs along with their interactions with the SARS-CoV-2 virus-host interactants. To achieve this, we first generated DEG sets in each individual study by randomly picking the

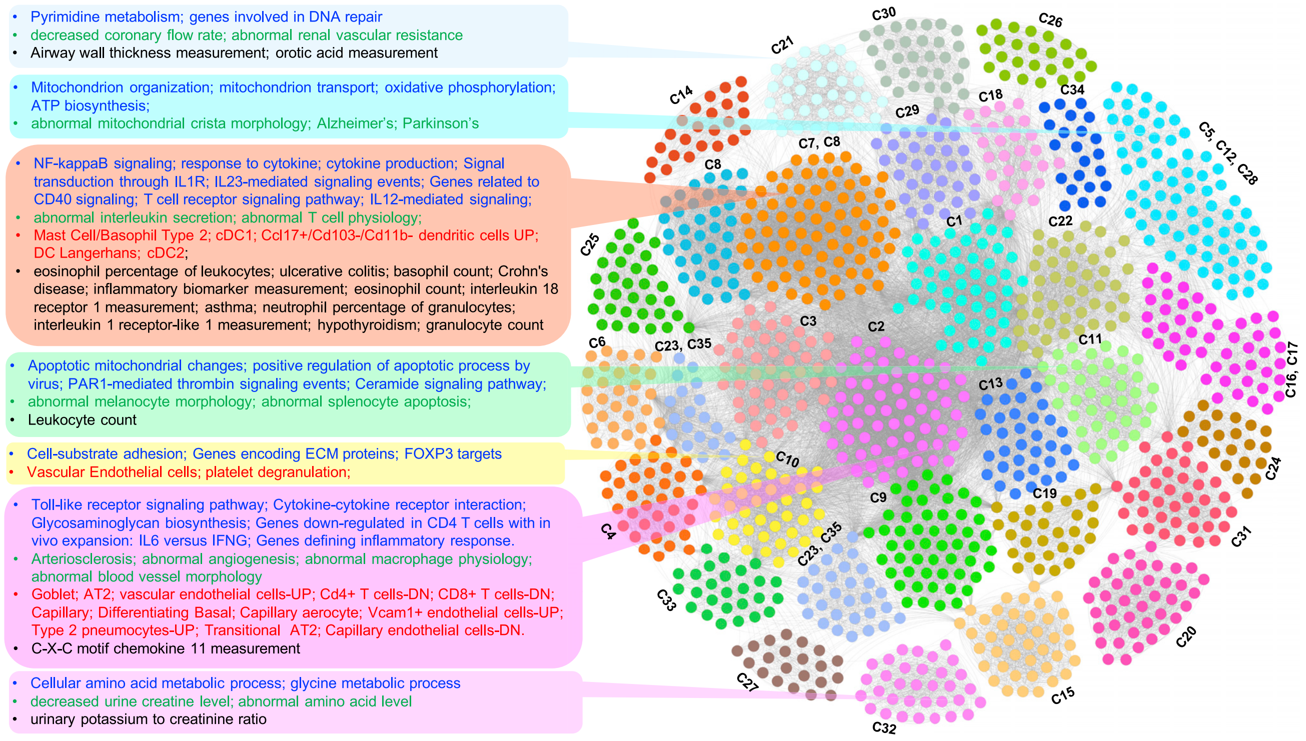

Figure 5. Network visualization of the results from joint analysis of multiple annotations from the 35 gene clusters

Network representation of clustered enriched terms from functional enrichment analysis (multiple annotations such as Gene Ontology, pathways, lung cell types, and GWA loci) of candidate gene clusters from the integrated consensus DEG and SARS-CoV-2-host interacting protein network. Enriched terms from different annotation categories are represented as nodes, while the edges represent shared gene clusters (35 select gene clusters). Representative terms in some of the clustered functional modules are listed on the left side with different font colors representing different annotation categories (blue, biological processes/pathways; green, phenotype; red, cell type; black, GWA trait). The underlying gene clusters (35 select gene clusters) for each of the clustered functional terms are also shown.

same number of genes as obtained originally (Table 1) and identified the consensus signature from among them. As is the case in our earlier experiments, we observed that the counts of consensus DEGs in our random trials are significantly lower than the observed gene sets (Figure S4C). These consensus genes were then combined with the SARS-CoV-2-human virus-host interactome (336 genes), and the integrated gene set was tested for enrichment of protein-protein interactions from STRING.[19] We repeated these two independent steps 1,000 different times and plotted the enrichment p values in each case (Figure S4C). On average, the consensus DEG counts from our random tests were around 300 genes while the empirical p values were less significant than the observed level ($p < 1.0 \times 10^{-16}$). Although we found statistically significant ($p \leq 0.05$) PPI enrichments in some of our trials, we hypothesized that these might be driven by the 336 SARS-CoV-2 interactants. Therefore, we tried to test this in our final set of experiments by randomly picking 1,803 genes (1,467 conserved + 336 SARS-CoV-2 interactants) and then checked for their PPI enrichments. This time, we observed fewer significant enrichments ($p \leq 0.05$) among 1,000 independent trials (Figure S4D). We also found that the average local clustering coefficient values (from STRING) in each trial were smaller than the actual value (0.42) (Figure S4D). In all our experiments, we used the STRING API (https://string-db.org/help/api/) to compute PPI enrichments and to retrieve the clustering coefficient scores.

Functional and human lung cell markers enrichment analysis

Functional enrichment for Gene Ontology biological processes, mouse phenotypes, pathways, and 4,872 immunologic[61] and 50 hallmark[62] gene sets from MSigDB[63] was done using the ToppGene suite[64] while the pathway enrichment analysis using the Elsevier Pathway Collection was done using Enrichr.[65] Additionally, to detect specific cell types potentially perturbed or affected in COVID-19, we intersected the DEGs and gene clusters from SARS-CoV-2 infection models with cell-type markers (FDR $p \leq 0.05$; $\log FC \geq 0.5$) from normal adult human lung.[20–22]

Genome-wide association trait enrichment analysis

For gene and phenotype trait association analysis, we used data from the NCBI's Phenotype-Genotype Integrator (PheGenI)[16] and the NHGRI-EBI GWAS catalog.[17] We used significant (1×10^{-5}) vulnerability loci of various human physiological traits, excluding all intergenic variants. Additionally, we also included child trait associations for the mapped traits from the GWAS catalog. The child terms for each trait were obtained by parsing the experimental factor ontology hierarchy.[66] We applied Fisher's exact test to find the enrichments.

SUPPLEMENTAL INFORMATION

Supplemental information can be found online at https://doi.org/10.1016/j.patter.2021.100247.

ACKNOWLEDGMENTS

This study was supported in part by National Institutes of Health grant 1UG3TR002612 and by the Cincinnati Children's Hospital Medical Center.

AUTHOR CONTRIBUTIONS

S.G. and A.G.J. conceived and initiated this study. S.G., M.S., and A.G.J. collected and analyzed data. S.G. and A.G.J. interpreted results from data. S.G., M.S., and A.G.J. edited the manuscript. S.G. and A.G.J. wrote the first draft.

Table 3. Enriched functional terms shared among the 35 candidate gene clusters from the integrated network of consensus DEGs of the three SARS-CoV-2 infection models and the SARS-CoV-2-host protein interaction map

Cluster	Enriched functional terms
C-7 and C-29	atypical NF-κB pathway; autoimmune disease
C-2, C-7, and C11	apoptotic mitochondrial changes; positive regulation of apoptotic process by virus; PAR1-mediated thrombin signaling events; ceramide signaling pathway; abnormal melanocyte morphology; abnormal splenocyte apoptosis; leukocyte count
C-8 and C-19	canonical NF-κB pathway; regulation of cytoplasmic translation
C-2, C-7, C-3, and C-25	genes regulated by NF-κB; circadian rhythm—mammal; genes upregulated in regulatory T (FOXP3$^+$) cells from B6 mice
C-18, C-2, and C-5	proliferating basal; proliferating macrophages; DNA replication; E2F transcription factor network
C-13, C-7, and C-2	OLR1$^+$ classical monocytes; neutrophil activation; β3 integrin cell surface interactions; liver inflammation; increased susceptibility to bacterial infection
C-1, C-7, and C-10	dendritic cells; proliferating macrophages; innate immune response; increased susceptibility to infection; platelet function tests; immune effector process
C-2 and C-3	adventitial fibroblasts; mesothelial; intermediate monocytes; mRNA metabolic process; abnormal heart ventricle morphology; genes upregulated in response to low oxygen levels
C-21	decreased coronary flow rate; abnormal renal vascular resistance; airway wall thickness measurement; orotic acid measurement
C-23 and C-35	plasma cells; peptide metabolic process; translational initiation; regulation of translation; mitochondrial gene expression and translation
C-8 and C-2	apoptosis; activation of innate immune response; NOD-like receptor signaling pathway; Toll-like receptor signaling pathway; TNF receptor signaling pathway; immune system disease; respiratory system disease
C-7	NF-κB signaling; response to cytokine; signal transduction through IL1R; IL23-mediated signaling events; genes related to CD40 signaling; T cell receptor signaling pathway; IL12-mediated signaling; abnormal interleukin secretion; abnormal T cell physiology; mast cell/basophil type 2; cDC1; Langerhans dendritic cells; cDC2; ulcerative colitis; Crohn’s disease; inflammatory biomarker measurement; asthma; hypothyroidism; granulocyte count
C-9 and C-2	cell-substrate adhesion; extracellular matrix; genes encoding collagen proteins; endothelial cells; AT1; fibroblasts; smooth muscle cells; myofibroblasts; intracerebral hemorrhage; Marfan syndrome; β-blocking agent use measurement
C-10 and C-2	genes encoding extracellular matrix; arterial vascular endothelial cells; smooth muscle cells; platelet degranulation; membrane fusion; vesicle fusion; VEGF and VEGFR signaling network
C-5, C-12, and C-28	mitochondrion organization; mitochondrion transport; oxidative phosphorylation; ATP biosynthesis; abnormal mitochondrial crista morphology; Alzheimer’s; Parkinson’s
C-16 and C-17	fatty acid catabolic process; peroxisome organization; lipid oxidation; propanoate metabolism; PPAR signaling pathway; abnormal lipid level; blood metabolite measurement

The shared terms (biological processes, pathways, cell types, and phenotypic traits) are found through meta-analysis of the enriched terms from different annotation categories for the 35 gene clusters. The complete network along with all enriched terms and cluster details are presented in Table S10. IL, interleukin; cDC1 and cDC2, conventional dendritic cell types 1 and 2; PPAR, peroxisome proliferator-activated receptor; TNF, tumor necrosis factor; VEGF, vascular endothelial growth factor; VEGFR, VEGF receptor.

DECLARATION OF INTERESTS

The authors declare no competing interests.

Received: December 21, 2020
Revised: January 18, 2021
Accepted: April 1, 2021
Published: April 5, 2021

REFERENCES

1. Spirin, V., and Mirny, L.A. (2003). Protein complexes and functional modules in molecular networks. Proc. Natl. Acad. Sci. U S A *100*, 12123–12128.
2. Barabasi, A.L., and Oltvai, Z.N. (2004). Network biology: understanding the cell’s functional organization. Nat. Rev. Genet. *5*, 101–113.
3. Ziegler, C.G.K., Allon, S.J., Nyquist, S.K., Mbano, I.M., Miao, V.N., Tzouanas, C.N., Cao, Y., Yousif, A.S., Bals, J., Hauser, B.M., et al. (2020). SARS-CoV-2 receptor ACE2 is an interferon-stimulated gene in human airway epithelial cells and is detected in specific cell subsets across tissues. Cell *181*, 1016–1035.e19.
4. Sungnak, W., Huang, N., Becavin, C., Berg, M., Queen, R., Litvinukova, M., Talavera-Lopez, C., Maatz, H., Reichart, D., Sampaziotis, F., et al. (2020). SARS-CoV-2 entry factors are highly expressed in nasal epithelial cells together with innate immune genes. Nat. Med. *26*, 681–687.
5. Cao, Y., Li, L., Feng, Z., Wan, S., Huang, P., Sun, X., Wen, F., Huang, X., Ning, G., and Wang, W. (2020). Comparative genetic analysis of the novel coronavirus (2019-nCoV/SARS-CoV-2) receptor ACE2 in different populations. Cell Discov, *6*, 11.
6. Consortium, G.T. (2015). Human genomics. The Genotype-Tissue Expression (GTEx) pilot analysis: multitissue gene regulation in humans. Science *348*, 648–660.
7. Hernandez Cordero, A.I., Li, X., Yang, C.X., Milne, S., Bosse, Y., Joubert, P., Timens, W., van den Berge, M., Nickle, D., Hao, K., et al. (2020). Gene

expression network analysis provides potential targets against SARS-CoV-2. Sci. Rep. *10*, 21863.

8. Guzzi, P.H., Mercatelli, D., Ceraolo, C., and Giorgi, F.M. (2020). Master regulator analysis of the SARS-CoV-2/human interactome. J. Clin. Med. *9*, 982.
9. Feng, Q., Li, L., and Wang, X. (2020). Identifying pathways and networks associated with the SARS-CoV-2 cell receptor ACE2 based on gene expression profiles in normal and SARS-CoV-2-infected human tissues. Front. Mol. Biosci. *7*, 568954.
10. Nadeau, R., Shahryari Fard, S., Scheer, A., Hashimoto-Roth, E., Nygard, D., Abramchuk, I., Chung, Y.-E., Bennett, S.A.L., and Lavallée-Adam, M. (2020). computational identification of human biological processes and protein sequence motifs putatively targeted by SARS-CoV-2 proteins using protein-protein interaction networks. J. Proteome Res. *19*, 4553–4566.
11. Ahmed, F. (2020). A network-based analysis reveals the mechanism underlying vitamin D in suppressing cytokine storm and virus in SARS-CoV-2 infection. Front. Immunol. *11*, 590459.
12. Blanco-Melo, D., Nilsson-Payant, B.E., Liu, W.C., Uhl, S., Hoagland, D., Moller, R., Jordan, T.X., Oishi, K., Panis, M., Sachs, D., et al. (2020). Imbalanced host response to SARS-CoV-2 drives development of COVID-19. Cell *181*, 1036–1045 e9.
13. Riva, L., Yuan, S., Yin, X., Martin-Sancho, L., Matsunaga, N., Pache, L., Burgstaller-Muehlbacher, S., De Jesus, P.D., Teriete, P., Hull, M.V., et al. (2020). Discovery of SARS-CoV-2 antiviral drugs through large-scale compound repurposing. Nature *586*, 113–119.
14. Sun, J., Zhuang, Z., Zheng, J., Li, K., Wong, R.L., Liu, D., Huang, J., He, J., Zhu, A., Zhao, J., et al. (2020). Generation of a broadly useful model for COVID-19 pathogenesis, vaccination, and treatment. Cell *182*, 734–743 e5.
15. Lieberman, N.A.P., Peddu, V., Xie, H., Shrestha, L., Huang, M.L., Mears, M.C., Cajimat, M.N., Bente, D.A., Shi, P.Y., Bovier, F., et al. (2020). In vivo antiviral host transcriptional response to SARS-CoV-2 by viral load, sex, and age. PLoS Biol. *18*, e3000849.
16. Ramos, E.M., Hoffman, D., Junkins, H.A., Maglott, D., Phan, L., Sherry, S.T., Feolo, M., and Hindorff, L.A. (2014). Phenotype-Genotype Integrator (PheGenI): synthesizing genome-wide association study (GWAS) data with existing genomic resources. Eur. J. Hum. Genet. *22*, 144–147.
17. Buniello, A., MacArthur, J.A.L., Cerezo, M., Harris, L.W., Hayhurst, J., Malangone, C., McMahon, A., Morales, J., Mountjoy, E., Sollis, E., et al. (2019). The NHGRI-EBI GWAS catalog of published genome-wide association studies, targeted arrays and summary statistics 2019. Nucleic Acids Res. *47*, D1005–D1012.
18. Gordon, D.E., Jang, G.M., Bouhaddou, M., Xu, J., Obernier, K., White, K.M., O'Meara, M.J., Rezelj, V.V., Guo, J.Z., Swaney, D.L., et al. (2020). A SARS-CoV-2 protein interaction map reveals targets for drug repurposing. Nature *583*, 459–468.
19. Szklarczyk, D., Gable, A.L., Lyon, D., Junge, A., Wyder, S., Huerta-Cepas, J., Simonovic, M., Doncheva, N.T., Morris, J.H., Bork, P., et al. (2019). STRING v11: protein-protein association networks with increased coverage, supporting functional discovery in genome-wide experimental datasets. Nucleic Acids Res. *47*, D607–D613.
20. Habermann, A.C., Gutierrez, A.J., Bui, L.T., Yahn, S.L., Winters, N.I., Calvi, C.L., Peter, L., Chung, M.-I., Taylor, C.J., Jetter, C., et al. (2020). Single-cell RNA sequencing reveals profibrotic roles of distinct epithelial and mesenchymal lineages in pulmonary fibrosis. Sci. Adv. *6*, eaba1972.
21. Adams, T.S., Schupp, J.C., Poli, S., Ayaub, E.A., Neumark, N., Ahangari, F., Chu, S.G., Raby, B.A., DeIuliis, G., Januszyk, M., et al. (2020). Single-cell RNA-seq reveals ectopic and aberrant lung-resident cell populations in idiopathic pulmonary fibrosis. Sci. Adv. *6*, eaba1983.
22. Travaglini, K.J., Nabhan, A.N., Penland, L., Sinha, R., Gillich, A., Sit, R.V., Chang, S., Conley, S.D., Mori, Y., Seita, J., et al. (2020). A molecular cell atlas of the human lung from single-cell RNA sequencing. Nature *587*, 619–625.
23. Lehnardt, S., Massillon, L., Follett, P., Jensen, F.E., Ratan, R., Rosenberg, P.A., Volpe, J.J., and Vartanian, T. (2003). Activation of innate immunity in the CNS triggers neurodegeneration through a Toll-like receptor 4-dependent pathway. Proc. Natl. Acad. Sci. U S A *100*, 8514–8519.
24. Godbout, J.P., Chen, J., Abraham, J., Richwine, A.F., Berg, B.M., Kelley, K.W., and Johnson, R.W. (2005). Exaggerated neuroinflammation and sickness behavior in aged mice following activation of the peripheral innate immune system. FASEB J. *19*, 1329–1331.
25. Elson, C.O., Cong, Y., McCracken, V.J., Dimmitt, R.A., Lorenz, R.G., and Weaver, C.T. (2005). Experimental models of inflammatory bowel disease reveal innate, adaptive, and regulatory mechanisms of host dialogue with the microbiota. Immunol. Rev. *206*, 260–276.
26. Fazzini, E., Fleming, J., and Fahn, S. (1992). Cerebrospinal fluid antibodies to coronavirus in patients with Parkinson's disease. Mov Disord. *7*, 153–158.
27. Troyer, E.A., Kohn, J.N., and Hong, S. (2020). Are we facing a crashing wave of neuropsychiatric sequelae of COVID-19? Neuropsychiatric symptoms and potential immunologic mechanisms. Brain Behav. Immun. *87*, 34–39.
28. Sims, A.C., Tilton, S.C., Menachery, V.D., Gralinski, L.E., Schafer, A., Matzke, M.M., Webb-Robertson, B.J., Chang, J., Luna, M.L., Long, C.E., et al. (2013). Release of severe acute respiratory syndrome coronavirus nuclear import block enhances host transcription in human lung cells. J. Virol. *87*, 3885–3902.
29. Regla-Nava, J.A., Nieto-Torres, J.L., Jimenez-Guardeno, J.M., Fernandez-Delgado, R., Fett, C., Castano-Rodriguez, C., Perlman, S., Enjuanes, L., and DeDiego, M.L. (2015). Severe acute respiratory syndrome coronaviruses with mutations in the E protein are attenuated and promising vaccine candidates. J. Virol. *89*, 3870–3887.
30. Totura, A.L., Whitmore, A., Agnihothram, S., Schafer, A., Katze, M.G., Heise, M.T., and Baric, R.S. (2015). Toll-like receptor 3 signaling via TRIF contributes to a protective innate immune response to severe acute respiratory syndrome coronavirus infection. mBio *6*, e00638-15.
31. Gordon, D.E., Hiatt, J., Bouhaddou, M., Rezelj, V.V., Ulferts, S., Braberg, H., Jureka, A.S., Obernier, K., Guo, J.Z., Batra, J., et al. (2020). Comparative host-coronavirus protein interaction networks reveal pan-viral disease mechanisms. Science *370*, eabe9403.
32. Bastian, M., Heymann, S., and Jacomy, M. (2009). Gephi: An Open Source Software for Exploring and Manipulating Networks. https://gephi.org/users/publications/%20.
33. Zhou, Y., Zhou, B., Pache, L., Chang, M., Khodabakhshi, A.H., Tanaseichuk, O., Benner, C., and Chanda, S.K. (2019). Metascape provides a biologist-oriented resource for the analysis of systems-level datasets. Nat. Commun. *10*, 1523.
34. Blondel, V.D., Guillaume, J.-L., Lambiotte, R., and Lefebvre, E. (2008). Fast unfolding of communities in large networks. J. Stat. Mech. Theor. Exp. *2008*, P10008.
35. Newman, M.E.J. (2004). Analysis of weighted networks. Phys. Rev. E *70*, 056131.
36. Manne, B.K., Denorme, F., Middleton, E.A., Portier, I., Rowley, J.W., Stubben, C., Petrey, A.C., Tolley, N.D., Guo, L., Cody, M., et al. (2020). Platelet gene expression and function in patients with COVID-19. Blood *136*, 1317–1329.
37. Hottz, E.D., Azevedo-Quintanilha, I.G., Palhinha, L., Teixeira, L., Barreto, E.A., Pao, C.R.R., Righy, C., Franco, S., Souza, T.M.L., Kurtz, P., et al. (2020). Platelet activation and platelet-monocyte aggregate formation trigger tissue factor expression in patients with severe COVID-19. Blood *136*, 1330–1341.
38. Huertas, A., Montani, D., Savale, L., Pichon, J., Tu, L., Parent, F., Guignabert, C., and Humbert, M. (2020). Endothelial cell dysfunction: a major player in SARS-CoV-2 infection (COVID-19)? Eur. Respir. J. *56*, 2001634.
39. Zheng, Y., and Rudensky, A.Y. (2007). Foxp3 in control of the regulatory T cell lineage. Nat. Immunol. *8*, 457–462.

40. Williams, L.M., and Rudensky, A.Y. (2007). Maintenance of the Foxp3-dependent developmental program in mature regulatory T cells requires continued expression of Foxp3. Nat. Immunol. *8*, 277–284.

41. Zheng, Y., Zhu, W., Haribhai, D., Williams, C.B., Aster, R.H., Wen, R., and Wang, D. (2019). Regulatory T cells control PF4/heparin antibody production in mice. J. Immunol. *203*, 1786–1792.

42. Bennett, C.L., Christie, J., Ramsdell, F., Brunkow, M.E., Ferguson, P.J., Whitesell, L., Kelly, T.E., Saulsbury, F.T., Chance, P.F., and Ochs, H.D. (2001). The immune dysregulation, polyendocrinopathy, enteropathy, X-linked syndrome (IPEX) is caused by mutations of FOXP3. Nat. Genet. *27*, 20–21.

43. Stephen-Victor, E., Das, M., Karnam, A., Pitard, B., Gautier, J.-F., and Bayry, J. (2020). Potential of regulatory T-cell-based therapies in the management of severe COVID-19. Eur. Respir. J. *56*, 2002182.

44. Hassan, A.O., Case, J.B., Winkler, E.S., Thackray, L.B., Kafai, N.M., Bailey, A.L., McCune, B.T., Fox, J.M., Chen, R.E., Alsoussi, W.B., et al. (2020). A SARS-CoV-2 infection model in mice demonstrates protection by neutralizing antibodies. Cell *182*, 744–753.e4.

45. Johansen, M.D., Irving, A., Montagutelli, X., Tate, M.D., Rudloff, I., Nold, M.F., Hansbro, N.G., Kim, R.Y., Donovan, C., Liu, G., et al. (2020). Animal and translational models of SARS-CoV-2 infection and COVID-19. Mucosal Immunol. *13*, 877–891.

46. Lamers, M.M., Beumer, J., van der Vaart, J., Knoops, K., Puschhof, J., Breugem, T.I., Ravelli, R.B.G., Paul van Schayck, J., Mykytyn, A.Z., Duimel, H.Q., et al. (2020). SARS-CoV-2 productively infects human gut enterocytes. Science *369*, 50–54.

47. Lamers, M.M., van der Vaart, J., Knoops, K., Riesebosch, S., Breugem, T.I., Mykytyn, A.Z., Beumer, J., Schipper, D., Bezstarosti, K., Koopman, C.D., et al. (2020). An organoid-derived bronchioalveolar model for SARS-CoV-2 infection of human alveolar type II-like cells. EMBO J. e105912.

48. Katsura, H., Sontake, V., Tata, A., Kobayashi, Y., Edwards, C.E., Heaton, B.E., Konkimalla, A., Asakura, T., Mikami, Y., Fritch, E.J., et al. (2020). Human lung stem cell-based alveolospheres provide insights into SARS-CoV-2-mediated interferon responses and pneumocyte dysfunction. Cell Stem Cell *27*, 890–904.e8.

49. Mulay, A., Konda, B., Garcia, G., Yao, C., Beil, S., Sen, C., Purkayastha, A., Kolls, J.K., Pociask, D.A., Pessina, P., et al. (2020). SARS-CoV-2 infection of primary human lung epithelium for COVID-19 modeling and drug discovery. bioRxiv. https://doi.org/10.1101/2020.06.29.174623.

50. Yue, Z., Zhang, E., Xu, C., Khurana, S., Batra, N., Dang, S.D.H., Cimino, J.J., and Chen, J.Y. (2021). PAGER-CoV: a comprehensive collection of pathways, annotated gene-lists and gene signatures for coronavirus disease studies. Nucleic Acids Res. *49*, D589–D599.

51. Kuleshov, M.V., Stein, D.J., Clarke, D.J.B., Kropiwnicki, E., Jagodnik, K.M., Bartal, A., Evangelista, J.E., Hom, J., Cheng, M., Bailey, A., et al. (2020). The COVID-19 drug and gene set library. Patterns (N Y) *1*, 100090.

52. Chen, Q., Allot, A., and Lu, Z. (2021). LitCovid: an open database of COVID-19 literature. Nucleic Acids Res. *49*, D1534–D1540.

53. Brohee, S., and van Helden, J. (2006). Evaluation of clustering algorithms for protein-protein interaction networks. BMC Bioinformatics *7*, 488.

54. Silverbush, D., Cristea, S., Yanovich, G., Geiger, T., Beerenwinkel, N., and Sharan, R. (2018). ModulOmics: integrating multi-omics data to identify cancer driver modules. bioRxiv, 288399.

55. Chaturvedi, P. (2018). Computational Suite for Bioinformaticians and Biologists (v3.0). https://github.com/praneet1988/Computational-Suite-For-Bioinformaticians-and-Biologists.

56. Andrews, S. (2010). FastQC: A Quality Control Tool for High Throughput Sequence Data. https://www.bioinformatics.babraham.ac.uk/projects/fastqc/.

57. Joint Genome Institute (2021). BBDuk Guide. https://jgi.doe.gov/data-and-tools/bbtools/bb-tools-user-guide/bbduk-guide/.

58. Li, B., and Dewey, C.N. (2011). RSEM: accurate transcript quantification from RNA-Seq data with or without a reference genome. BMC Bioinformatics *12*, 323.

59. Risso, D., Ngai, J., Speed, T.P., and Dudoit, S. (2014). Normalization of RNA-seq data using factor analysis of control genes or samples. Nat. Biotechnol. *32*, 896–902.

60. Robinson, M.D., McCarthy, D.J., and Smyth, G.K. (2009). edgeR: a Bioconductor package for differential expression analysis of digital gene expression data. Bioinformatics *26*, 139–140.

61. Godec, J., Tan, Y., Liberzon, A., Tamayo, P., Bhattacharya, S., Butte, A.J., Mesirov, J.P., and Haining, W.N. (2016). Compendium of immune signatures Identifies conserved and species-specific biology in response to inflammation. Immunity *44*, 194–206.

62. Liberzon, A., Birger, C., Thorvaldsdottir, H., Ghandi, M., Mesirov, J.P., and Tamayo, P. (2015). The Molecular Signatures Database (MSigDB) hallmark gene set collection. Cell Syst *1*, 417–425.

63. Subramanian, A., Tamayo, P., Mootha, V.K., Mukherjee, S., Ebert, B.L., Gillette, M.A., Paulovich, A., Pomeroy, S.L., Golub, T.R., Lander, E.S., et al. (2005). Gene set enrichment analysis: a knowledge-based approach for interpreting genome-wide expression profiles. Proc. Natl. Acad. Sci. U S A *102*, 15545–15550.

64. Chen, J., Bardes, E.E., Aronow, B.J., and Jegga, A.G. (2009). ToppGene Suite for gene list enrichment analysis and candidate gene prioritization. Nucleic Acids Res. *37*, W305–W311.

65. Kuleshov, M.V., Jones, M.R., Rouillard, A.D., Fernandez, N.F., Duan, Q., Wang, Z., Koplev, S., Jenkins, S.L., Jagodnik, K.M., Lachmann, A., et al. (2016). Enrichr: a comprehensive gene set enrichment analysis web server 2016 update. Nucleic Acids Res. *44*, W90–W97.

66. Malone, J., Holloway, E., Adamusiak, T., Kapushesky, M., Zheng, J., Kolesnikov, N., Zhukova, A., Brazma, A., and Parkinson, H. (2010). Modeling sample variables with an experimental factor ontology. Bioinformatics *26*, 1112–1118.

Patterns, Volume *2*

Supplemental information

Secondary analysis of transcriptomes of SARS-CoV-2 infection models to characterize COVID-19

Sudhir Ghandikota, Mihika Sharma, and Anil G. Jegga

SUPPLEMENTAL ITEMS

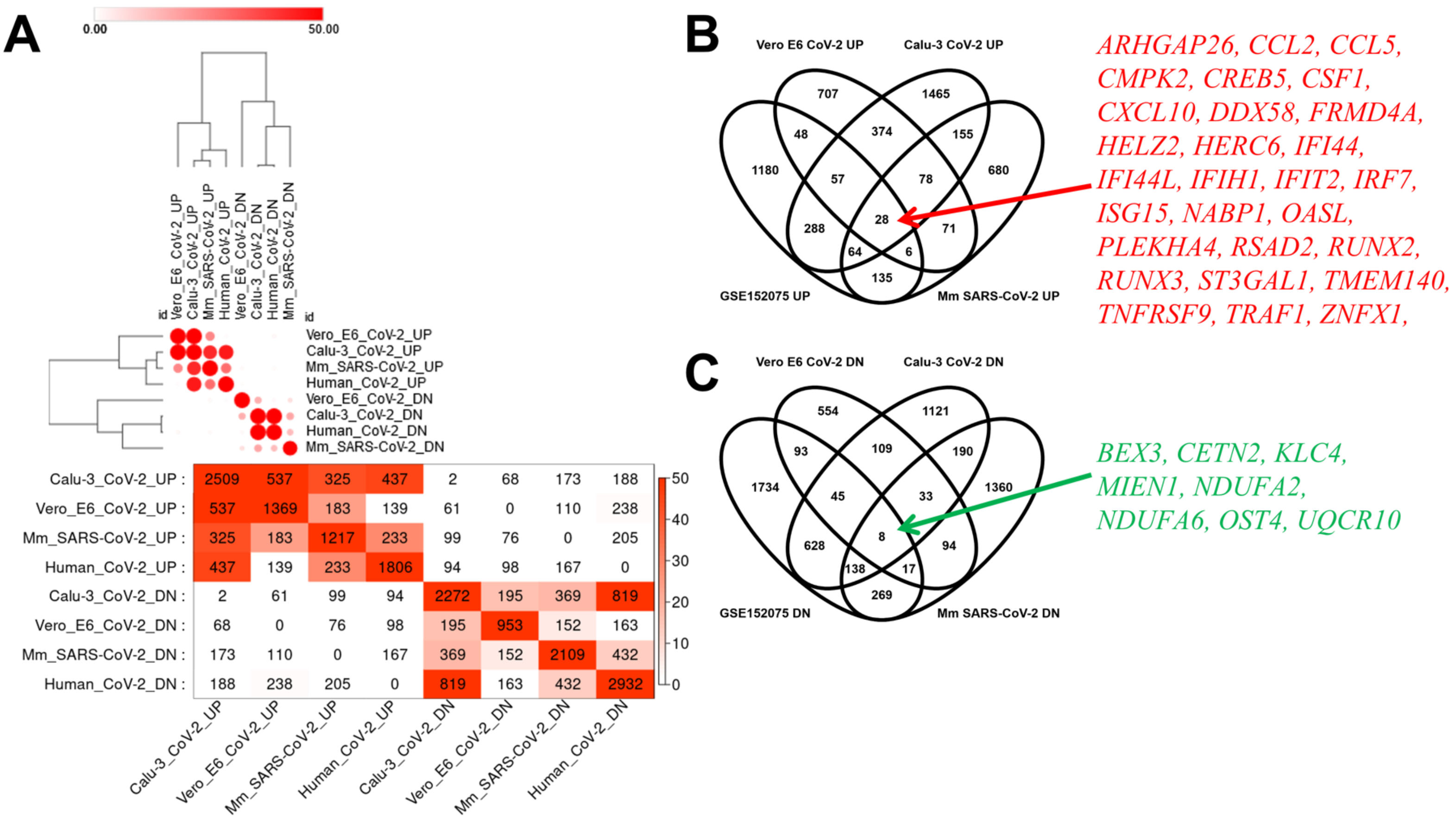

Figure S1: Transcriptomic overlap between SARS-CoV-2 infection models with that of DEGs from human nasopharyngeal swabs of COVID-19 patients. (A) Heatmap indicating the transcriptomic overlaps between the different SARS-CoV-2 infection models including the DEGs identified in nasopharyngeal swabs of human COVID-19 patients (GSE152075). **(B)** and **(C).** Venn diagrams showing intersections between up- or down-regulated DEGs respectively from the 3 SARS-CoV-2 models and the DEGs from nasopharyngeal swabs from human COVID-19 patients. There were 28 upregulated (Panel B) and 8 downregulated (Panel C) genes common to all.

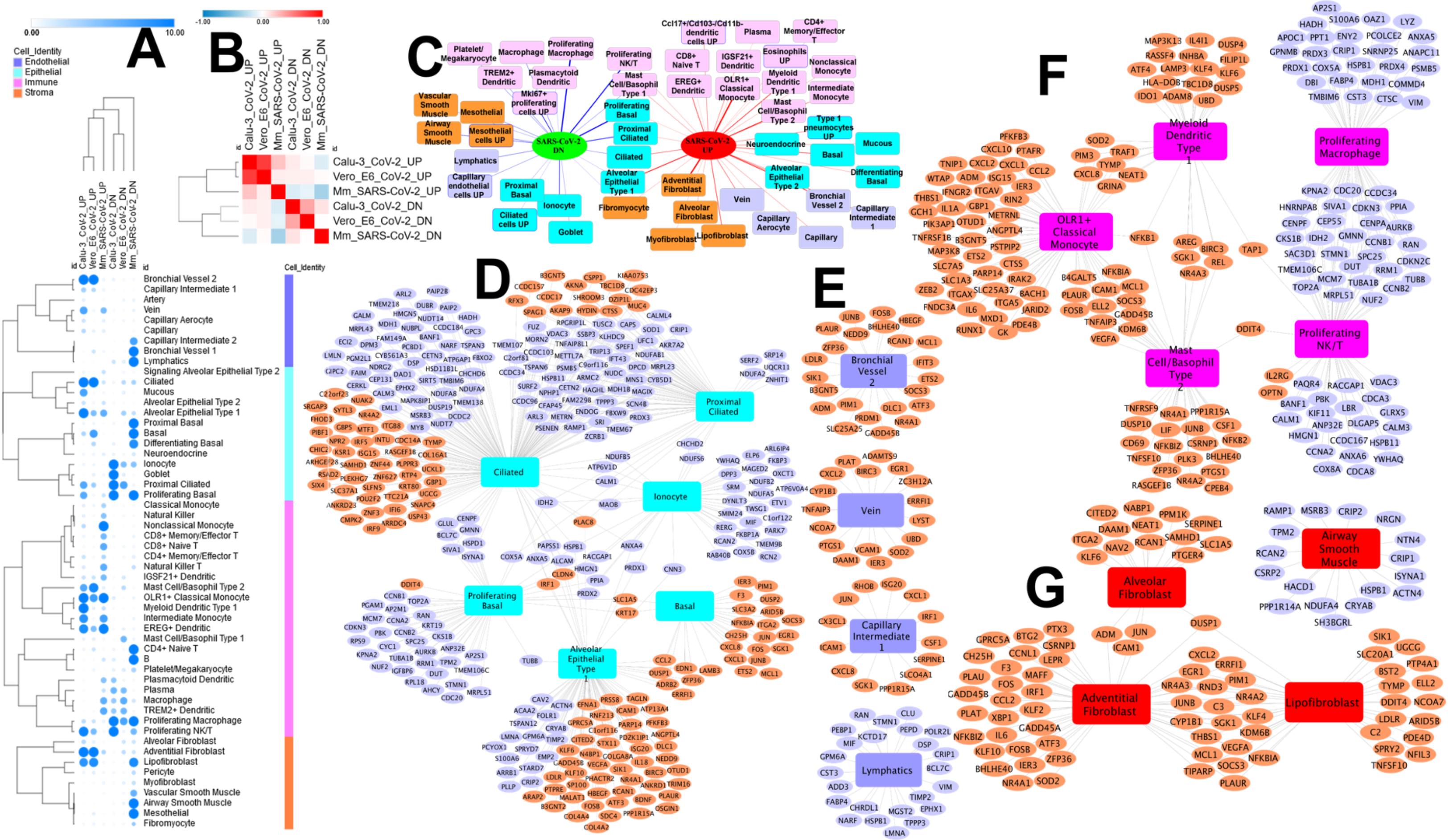

Figure S2: Lung cell type marker enrichment in consensus differentially expressed genes from the 3 SARS-CoV-2 infection models. **(A)** Enrichment heatmaps of single cell markers from normal human lung 22 among the differentially expressed genes from the 3 models of SARS-CoV-2 infection. The size and intensity of the colors in the circles is proportional to the significance of enrichment as measured by Fisher's exact test (negative log p-values). **(B)** Overlap heatmap of differentially expressed genes among the three SARS-CoV-2 infection models. **(C)** Network of enriched cell types from normal human lung 22 in the consensus differentially expressed genes from the 3 models of SARS-CoV-2 infection. The different colored rectangles are various cell types. The pink colored rectangles are myeloid cell types, purple-colored ones are lymphoid, green-colored ones are endothelial, blue-colored rectangles are epithelial, and the orange-colored nodes are stromal cell types. The width of the edges is proportional to the significance of the cell type enrichment (negative log p-value) measured by Fisher's exact test. **(D)-(G)** Network representations of enriched cell types and associated consensus differentially expressed genes from the 3 SARS-CoV-2 infection models. Consensus upregulated genes are in orange while the downregulated genes are in purple. The different panels represent epithelial, myeloid, lymphoid, endothelial, and stromal cell type enrichment networks along with their associated genes.

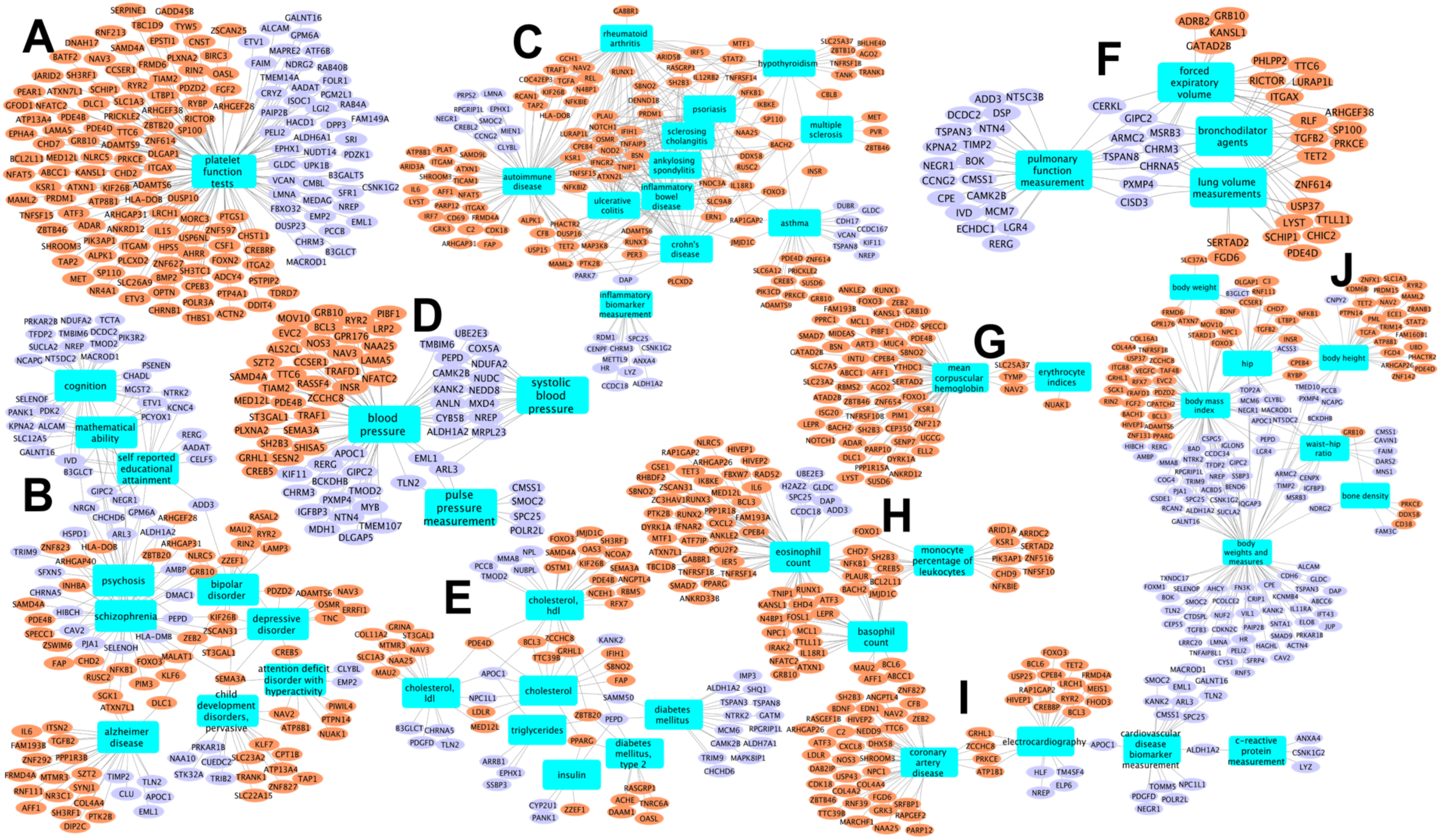

Figure S3: GWA loci enrichment in consensus differentially expressed genes from the 3 SARS-CoV-2 infection models. Network representation of select enriched PheGenI/GWA traits and their associated consensus DEGs from the 3 models of SARS-CoV-2 infection. Upregulated genes are in orange, downregulated genes are in purple, and the enriched traits are shown as blue colored rectangles. Networks are shown as different panels (A-J) based on their broad categorization, where possible. For instance, panel a shows immune system disorders, while panel g represents lung function measurements.

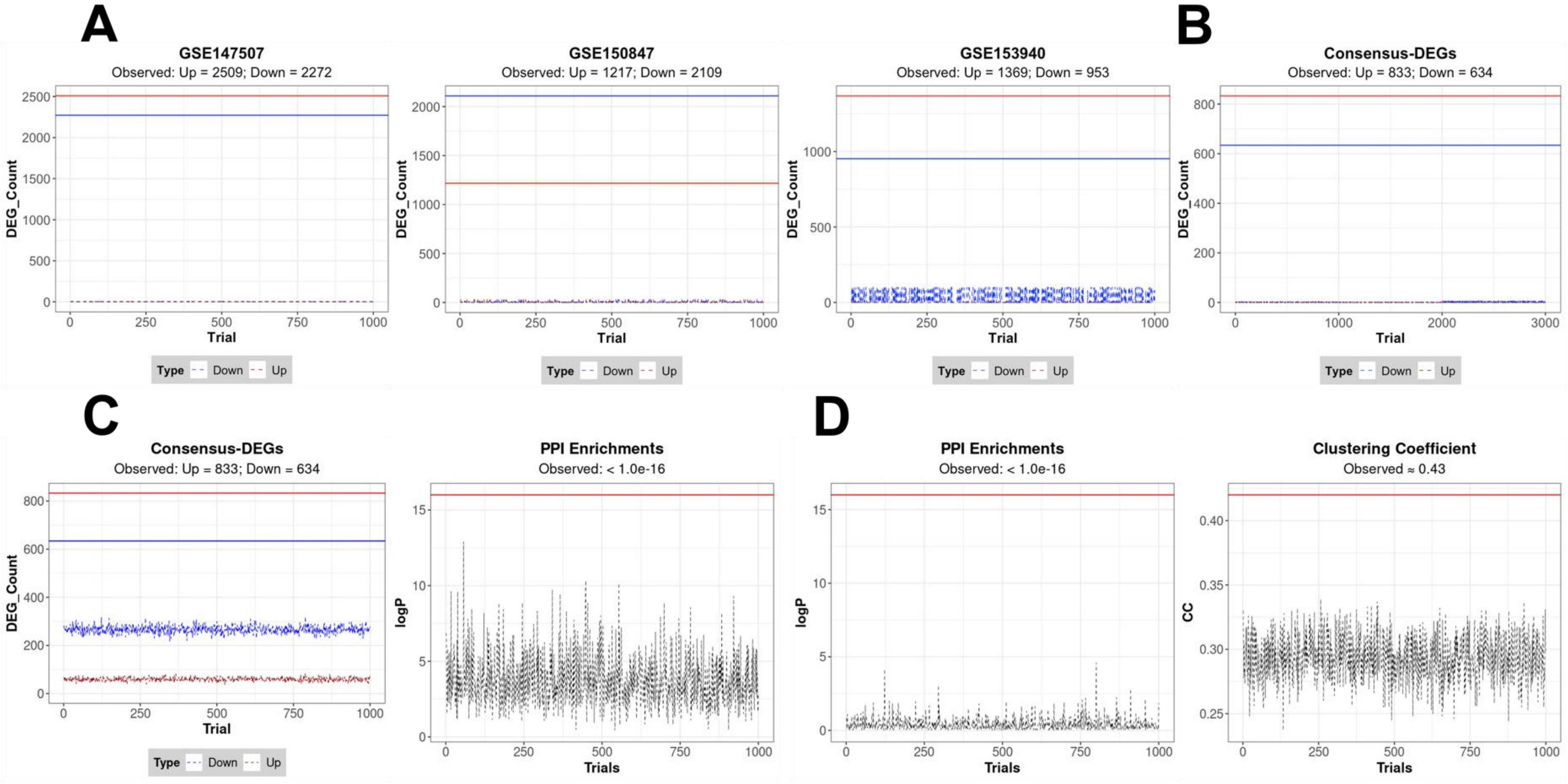

Figure S4: Robustness tests to validate the DEGs from the 3 SARS-CoV-2 infection models. **(A).** Plots of observed DEG counts in 1000 random trials where phenotype labels in each study were randomly permuted and DEGs were obtained based on the randomized labels. The actual counts of DEGs from each of the three SARS-CoV-2 infection models are indicated at the top of the plots below the GEO data set IDs. **(B).** Plots of consensus DEGs obtained by randomly permuting the phenotype labels from two of the three studies. This process was repeated 1000 times for each combination (3000 trials in total) and consensus DEGs were identified in each trial. **(C).** Plots of consensus DEG counts and PPI enrichments (negative log p-values) in randomized experiments where the DEGs were picked randomly from each model and used to identify the consensus candidates. The DEGs were then combined with the SARS-CoV-2 human interactants (336 genes) and tested for PPI enrichments. These two steps were repeated 1000 different times and the final consensus DEG counts (left) and PPI enrichment p-values (right) were recorded in each step. Actual DEG counts (upregulated = red; downregulated = blue) and the observed PPI significance level (< 1.0e-16) are represented as horizontal lines within each plot **(D).** Final set of randomized experiments where both the consensus DEGs (1467 genes) and the SARS-CoV-2 virus-host interactants (336 genes) are randomly generated in each trial. In addition to the PPI enrichment p-values (left), we also retrieved and plotted the average clustering coefficient values (right) in each trial.
